# An Assessment of the Environmental Impact of Construction Materials of Monocrystalline and Perovskite Photovoltaic Power Plants Toward Their Sustainable Development

**DOI:** 10.3390/ma17235787

**Published:** 2024-11-26

**Authors:** Izabela Piasecka, Zbigniew Kłos

**Affiliations:** 1 Faculty of Mechanical Engineering, Bydgoszcz University of Science and Technology, al. Prof. S. Kaliskiego 7, 85-796 Bydgoszcz, Poland; 2 Faculty of Civil Engineering and Transport, Poznań University of Technology, 60-965 Poznań, Poland; zbigniew.klos@put.poznan.pl

**Keywords:** environmental impact, structural materials, renewable energy materials, sustainable development, photovoltaic power plant

## Abstract

The interest in alternative energy sources, including the use of solar radiation energy, is growing year by year. Currently, the most frequently installed photovoltaic modules are made of single-crystalline silicon solar cells (sc-Si). However, one of the latest solutions are perovskite solar cells (PSC), which are considered the future of photovoltaics. Therefore, the main objective of this research was to assess the environmental impact of the construction materials of monocrystalline and perovskite photovoltaic power plants toward their sustainable development. The research object was the construction materials and components of two 1 MW photovoltaic power plants: one based on monocrystalline modules and the other on perovskite modules. The life cycle assessment (LCA) method was used for the analyses. The IMPACT World+, IPCC and CED models were used in it. The analyses were performed separately for five sets of elements: support structures, photovoltaic panels, inverter stations, electrical installations and transformers. Two post-consumer management scenarios were adopted: storage and recycling. The life cycle of a photovoltaic power plant based on photovoltaic modules made of perovskite cells is characterized by a smaller negative impact on the environment compared to traditional power plants with monocrystalline silicon modules. Perovskites, as a construction material of photovoltaic modules, fit better into the main assumptions of sustainable development compared to cells made of monocrystalline silicon. However, it is necessary to conduct further work which aims at reducing energy and material consumption in the life cycles of photovoltaic power plants.

## 1. Introduction

**Figure 1:**
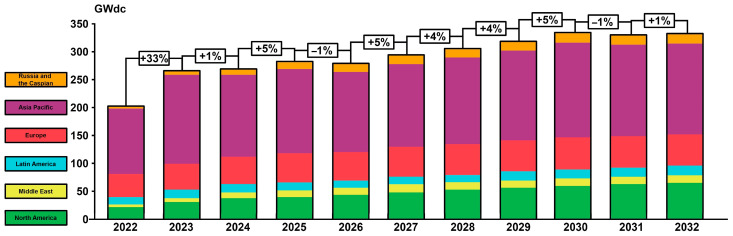
Forecast of growth in photovoltaic investments until 2032. Own work based on [4].

The photovoltaic industry is currently experiencing an intensive period, which continues to develop dynamically, bringing innovation, growth in installed capacities and strengthening the role of solar energy in the global energy mix. The photovoltaic sector is undergoing not only legislative changes, but also market and technological ones. Industry 4.0 tools are playing increasingly important roles in the photovoltaic industry—from big data to AI. It is predicted that the world will see further, significant development of photovoltaics in the coming years. By 2032, the capacities of the photovoltaic installations put into service each year are to be higher by an average of over 5% than the previous year. The reasons for such rapid development of the industry are seen in ambitious renewable energy goals, an increase in electrification, phasing out coal-fired power plants, concerns about energy security, expanding political support and falling costs of energy production from photovoltaics. According to forecasts, China is to remain the leader in photovoltaic investments for the coming years. This situation will not be changed by incentives for investment in the US, Europe and India. However, China’s current huge advantage in photovoltaic investments is expected to decrease due to the larger scale of investment in the US. (Figure 1) [1,2,3,4].

Solar energy is considered “green” and environmentally friendly. However, each source of energy, to a greater or lesser extent, affects the environment. Considering the life cycle of photovoltaic systems, they do not generate significant harmful effects on the environment during the exploitation stage. However, the stages of production and post-consumer management of their materials and components may potentially constitute sources of dangerous impacts, resulting in a decrease in the quality of the environment, an impact on human health and depletion of non-renewable raw materials [5,6,7].

There is a lack of research in the literature on life cycle analyses of photovoltaic power plants. Most often, one can find studies focusing on their key elements, like photovoltaic modules, and, in particular, on the various types of materials from which they are manufactured. Most research works contain analyses of the life cycles of cells and photovoltaic modules manufactured from silicon. Examples of such studies include Wang et al. [8], Kato et al. [9], Golroudbary et al. [10], Dones and Frischknecht [11], Heath et al. [12], Fthenakis and Kim [13], Reich et al. [14], Frankl [15] and Alsema [16], which are dedicated to single-crystalline silicon (sc-Si) modules. Other noteworthy research on these analyses includes studies conducted by Oliver and Jackson [17], Nomura [18], Kato [19], Ito [20,21], Dones and Frischknecht [11], Fthenakis and Kim [13], Fthenakis and Alsema [22] and Alsema [16], which analyze the life cycles of multi-crystalline silicon (mc-Si) modules. There have also been several studies on amorphous-silicon (a-Si) modules; for example, Kato [19], Ito [20] and Alsema [16], while other works, such as Bravi [23], discuss photovoltaic cells made of multi-junction thin-film silicon (µc-Si). There are also individual works devoted not only to silicon cells but also to those made of other materials; for example, studies by Ito [20], Fthenakis and Kim [13] and Fthenakis and Alsema [22] analyzed the life cycle of cadmium telluride (CdTe) modules. The topic of copper-indium-gallium-diselenide solar cells (CIGS) was taken up by Bravi [23], and the topic of dye-sensitized solar cells (DSSC) was taken up by Greijer [24].

However, there are also several studies on the life cycle of high-power photovoltaic systems in the literature. Examples include the analyses by Kato [9,25] covering 10, 30 and 100 MW power plants built from mc-Si, a-Si and CdTe cells, or the work by Schaefer and Hagedorn [26], which includes studies on 2.5 MW power plants built from sc-Si, mc-Si and a-Si cells.

Currently, the most commonly used type of photovoltaic module is made of monocrystalline silicon (sc-Si). However, research is constantly being conducted to find new solutions that are more efficient in terms of energy, economy and environment. One of them is perovskite modules (PSC), which, as the name suggests, contain a compound with a perovskite structure. Most often, it is a hybrid, organic-inorganic material based on lead or tin halide (the active layer that collects light). Perovskite materials are relatively cheap and easy to produce. The efficiency of this type of cell has increased from approx. 3.8% (2009) to approx. 25.5% (currently) in the case of systems with a single junction, and to approx. 28.8%, in the case of tandem cells based on silicon. For this reason, a decision was made to conduct a comparative analysis of the materials and structural components of two 1 MW photovoltaic power plants—the first one based on monocrystalline silicon modules and the second one—on perovskite modules [1,27,28,29].

Among the studies that assess the environmental impact of the life cycle of photovoltaic systems, there are no studies which conduct analyses using the relatively new IMPACT World+ method. Most of the studies conducted focus exclusively on the impact of global warming potential (GWP); for example, [19,20,21,22,23,24,25,26]. Usually, scientific studies do not consider a wider range of impacts that threaten human health, cause a decrease in the quality of ecosystems or deepen the depletion of raw materials. However, it was recognized that they required detailed analyses, especially in the context of the main assumptions of the sustainable development of the photovoltaic sector. 

Therefore, the aim of the study was to assess the environmental impact of construction materials of monocrystalline and perovskite photovoltaic power plants toward their sustainable development.

## 2. Materials and Methods

### 2.1. Object and Plan of Analysis

**Figure 2:**
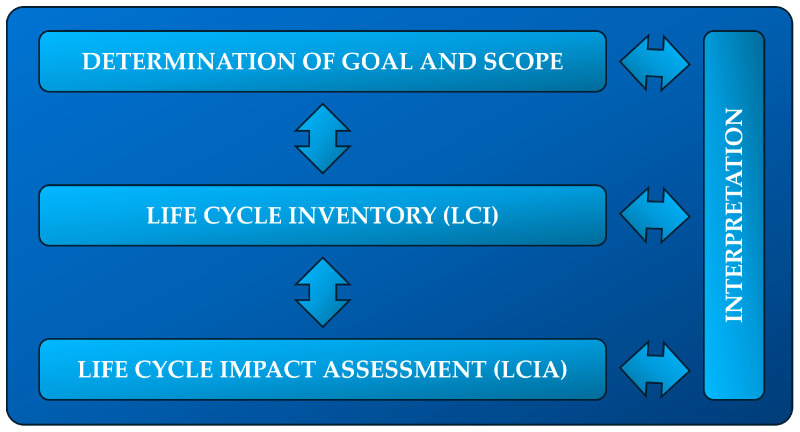
LCA stages of a photovoltaic power plant.

The subjects of the study were the materials and structural elements of two 1 MW photovoltaic power plants. The first one was based on modules made of monocrystalline silicon, while the second one was based on perovskites (tandem type, based on silicon). The life cycle assessment (LCA) method was selected to assess the environmental impact of the structural materials of monocrystalline and perovskite photovoltaic power plants toward their sustainable development. Based on the ISO 14040 [30] (environmental management, life-cycle assessment, principles and framework) and the ISO 14044 [31] (environmental management, life-cycle assessment, requirements and guidelines) standards, it was decided that the life cycle analysis in this study would include four consecutive stages: determination of goals and scope, life-cycle inventory (LCI), life-cycle impact assessment (LCIA) and interpretation (Figure 2) [32,33,34,35,36].

In the first step of the analyses, the aim and scope of the research were described (details are presented in Section 2.2). The analysis of the current state of knowledge and technology (Section 1) revealed that the literature lacks detailed studies on the assessment of the life cycle of photovoltaic power plants, including the impact of the construction materials of monocrystalline and perovskite power plants on the environment (considering not only CO_2_ emissions, but also other impacts on the environment, human health and depletion of raw materials). When the aim and scope of the research was developed, it was very important to collect as much reliable data as possible on the technical objects under study. This was made possible via cooperation with companies producing materials and components for photovoltaic power plants and dealing with their operational and post-consumer management (details in Section 2.3). The next stage of the research included a detailed analysis of the life cycle of the power plants under consideration. It was performed using SimaPro 9.5 software (Ecoinvent 3.9.1), IMPACT World+, IPCC and CED models (details in Section 2.4). The obtained results and their interpretation are presented in Section 3 and Section 4.

### 2.2. Determination of Goals and Scope

The first stage of the LCA analysis involves defining its purpose and scope. In this stage, decisions are made about the analytical models to be used, the boundaries of the system being studied are determined and the type of data that must be collected and analyzed is determined. In this phase, it is also necessary to analyze the relationships between the research object and the environment (considering all individual processes). Therefore, during this stage, it is crucial to define the product system (in this case, the photovoltaic power plant), the functional unit and the boundaries of the system [37,38,39,40].

The main objective of the conducted analyses was to compare the impact of construction materials occurring in the life cycle of a photovoltaic power plant based on monocrystalline silicon modules and perovskite modules on the environment (comparative analysis). The LCA analysis was conducted to identify potential differences in the size of the environmental impact of two photovoltaic power plants, the construction of which was based on two different technologies (sc-Si and PSC).

The systems of the studied power plants were built in such a way that they were comparable in terms of the breadth and depth of the analyses conducted. The companies with whom cooperation was established and which provided the data necessary for the research had a strong position on the European market, hence, the geographical horizon included the area of Europe. It was assumed that all materials, components and electrical energy necessary for the processes occurring in the life cycle were produced in this area. The time horizon for both facilities was also the same and amounted to 20 years. This time period corresponds to the recommendations of photovoltaic module manufacturers regarding the appropriate time of their operation and is consistent with the practical experience of photovoltaic companies in this area (reduction in energy conversion efficiency over time). Transport processes were not taken into account in the analyses, due to the possibility of large differences in this respect, depending on the potential location of the production plant and the area of assembly and subsequent operation of the test objects. In all analyses, the cut-off level was equal to 0.1%.

The conducted studies were classified as bottom-up analyses, which were used, on the one hand, to describe the existing reality (retrospective analysis), and, on the other hand, to model more sustainable construction solutions (prospective analysis). Due to the high level of advancement, the conducted studies can be classified as detailed analyses. The data used were obtained directly from the manufacturers and companies involved in the operational and post-consumer management of the studied photovoltaic power plants, or when this was not possible—from SimaPro software databases. The installed capacity of both technical facilities, 1 MW, was assumed as the functional unit. The environmental assessment included 27 impact categories of the IMPACT World+ model, 3 impact categories of the IPCC model and 6 impact categories of the CED model. The results were also grouped into two impact areas characterizing the impact on human health and the quality of ecosystems [41,42,43,44,45].

One of the basic research assumptions was to conduct the most detailed analyses possible, covering as many areas of impact on the environment as possible, both in terms of the impact on human health, environmental quality and the issue of resource depletion. For this reason, it was decided to use the IMPACT World+ model (27 categories), which, alongside ReCiPe 2016 (22 categories), stands out with the largest number of available impact categories. An important aspect was also the possibility of assessing the potential impact on the environment in two time perspectives: short term and long term. Most life cycle analyses presented in the literature include the impact of photovoltaic modules on the global warming potential. In order to compare the results presented in this study with the results found in the literature, a decision was made to conduct additional analyses using the latest LCA model in this area—IPCC 2021. Nowadays, the issue of energy consumption is also an extremely important aspect, which may seem particularly interesting in relation to technical facilities that produce energy. For this reason, it was decided to conduct analyses with the CED model intended for this purpose.

### 2.3. Life Cycle Inventory (LCI)

The second stage of the assessment includes data collection and their preliminary analysis. Quantitative data are collected to determine the set of inputs and outputs of the examined facility. This is an activity necessary to achieving the purpose of the analysis and to creating a model of the life cycle of the photovoltaic power plants considered. During this phase, the collected input data (energy and material) and output streams (waste and emissions) are identified and quantified. The collected information must also be analyzed for its reliability, completeness and accuracy [46,47,48].

All processes that took place in the life cycle of the assessed photovoltaic power plants were interconnected by energy and matter flows. Data collected during the LCI were assigned to individual unit processes, to verify them later (based on the mass and energy balance). Models were systematically constructed and then filled with data, with the size of input data always corresponding to the size of output data. The input data included main and auxiliary materials and water demand. The output data included emissions and main products. The data on key processes were obtained by cooperating with the manufacturers and companies involved in operational and post-consumer management. A small amount of data on less important materials and processes (in terms of their impact on the environment) was downloaded from SimaPro 9.5 software (database: Ecoinvent 3.9.1). Due to the conclusion of a confidentiality agreement with the companies producing, operating and managing the photovoltaic power plants, this study does not disclose technological data or information on the structure of these facilities in detail [49,50,51,52,53].

**Figure 3:**
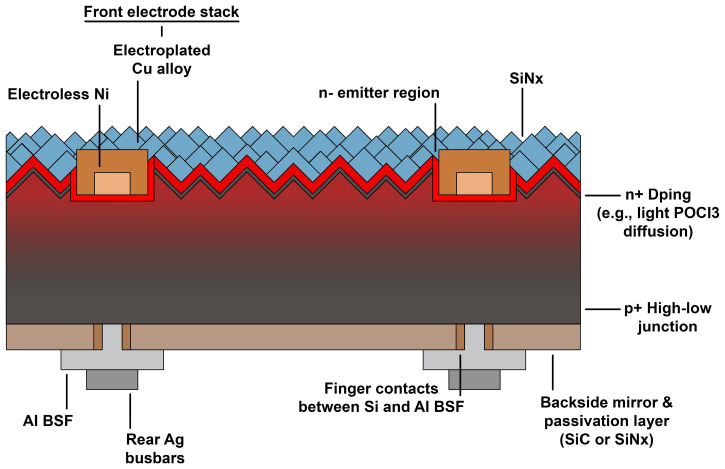
Structure of monocrystalline solar cell (sc-Si). Own work based on [54].

**Figure 4:**
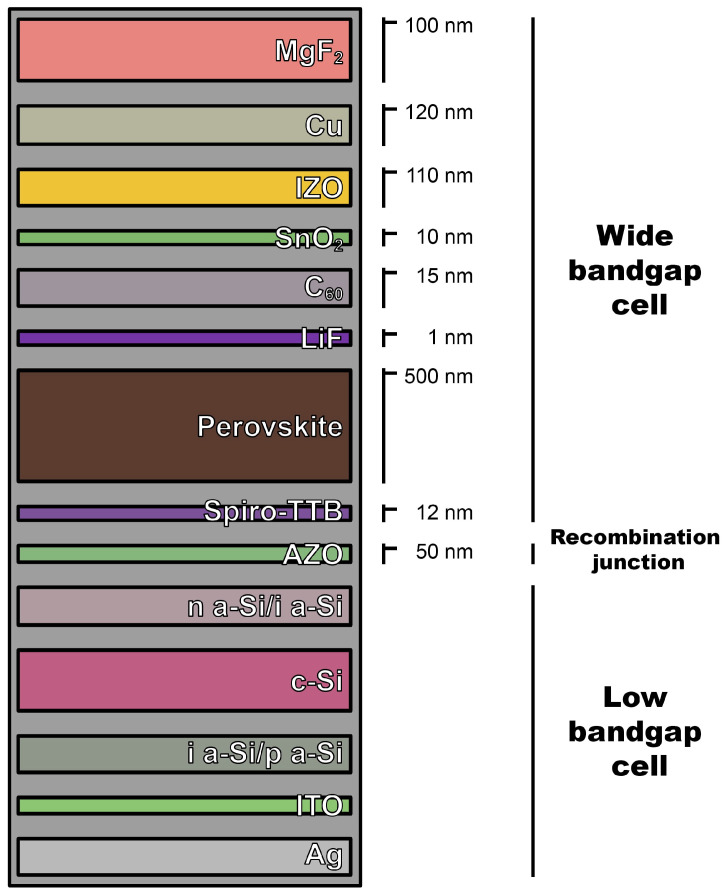
Schematic of perovskite-silicon tandem solar cell. Own work based on [55].

Silicon is a material that is used in the production of both monocrystalline and perovskite cells. Monocrystalline silicon cells are made using the energy-intensive Czochralski method. Their efficiency is higher compared to other silicon cells, such as polycrystalline or amorphous (approx. 22%). A single monocrystalline photovoltaic cell consists of a silicon wafer. On the upper surface of the wafer, an electron-collecting electrode in the form of a grid is placed, and on the lower surface, a lower electrode in the form of a metallic layer is applied (Figure 3). The efficiency of perovskite-silicon tandem solar cells is higher than that of single-crystalline silicon solar cells (approx. 25%). In the analyzed case, the low-bandgap cell took the form of a silicon heterojunction (SHJ) cell, while the wide-bandgap cell took the form of a (Cs, FA) Pb (I, Br)_3_ perovskite solar cell (Figure 4) [54,55].

**Figure 5:**
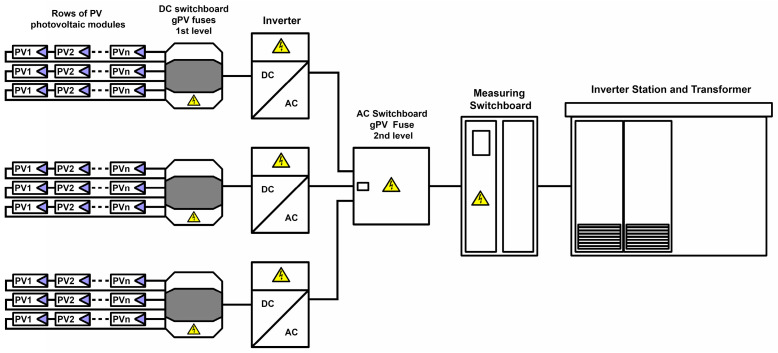
Functional block diagram of a photovoltaic power plant.

Both analyzed power plants had an installed capacity of 1 MW and consisted of the same five groups of structural element assemblies: photovoltaic panels, support structure, inverter station, electrical installation and transformer. The photovoltaic panels were embedded in the ground using a double piling system (two bases). Both solutions used central inverter stations, which constitute a complete solution dedicated to photovoltaic power plants. It houses all the electrical devices necessary for the quick connection of the power plant to the medium-voltage power grid. It includes two central inverters, an optimized transformer, a medium-voltage switchboard, a monitoring system and constant voltage connections for photovoltaic panels. The monocrystalline power plant was built using 2632 PV modules with a total surface area of 4896 m^2^. These were modules made of monocrystalline silicon (sc-Si) with a power of 380 W each, dimensions of 1769 × 1052 × 30 mm (1.86 m^2^) and a number of cells of 120 pieces per module. In the case of the perovskite power plant, there were 5556 PV modules with a total surface area of 4390 m^2^. These were silicon-based tandem cells (PSC) with a power of 180 W each and dimensions of 1245 × 635 × 2.8 mm (0.79 m^2^). Figure 5 shows a functional block diagram of the photovoltaic power plant (data obtained from the investor and manufacturers).

**Figure 6:**
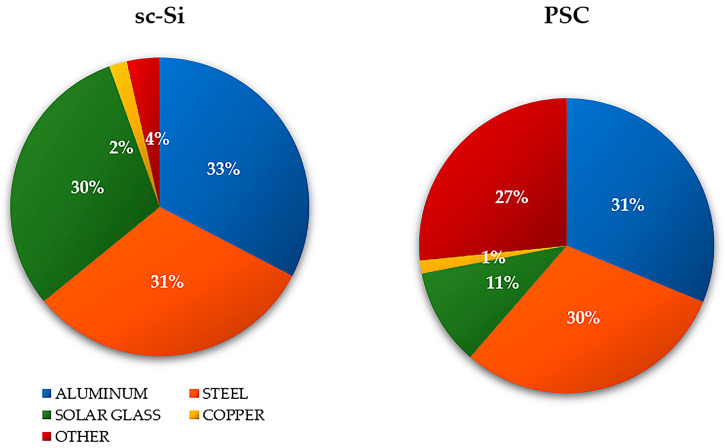
Mass percentage of the most important construction materials of a photovoltaic power plant based on monocrystalline (sc-Si) and perovskite (PSC) modules.

**Figure 7:**
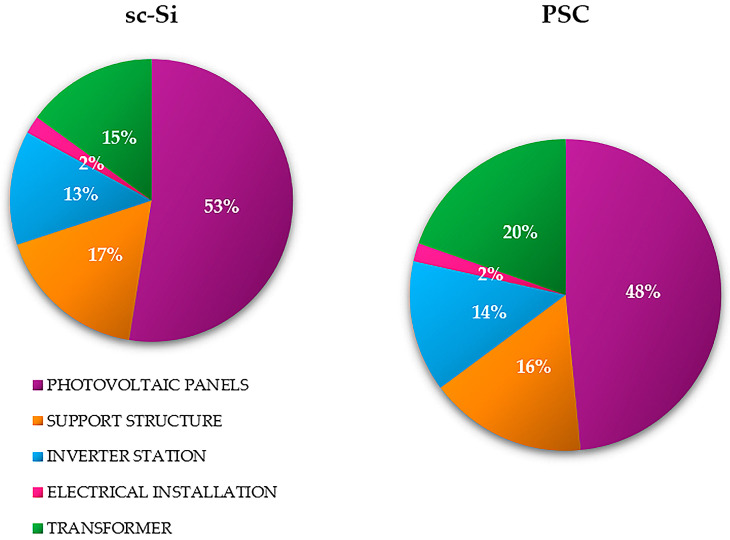
Mass percentage of the most important structural elements of a photovoltaic power plant based on monocrystalline (sc-Si) and perovskite (PSC) modules.

The total mass of the construction materials of the analyzed photovoltaic power plant based on monocrystalline modules (sc-Si) was about 200 tons; the total mass of the construction materials based on perovskite modules (PSC) was about 160 tons. The largest share in the mass of the sc-Si power plant was monocrystalline photovoltaic panels made of silicon—about 53% (of which about 47% was solar glass and about 45% was aluminum). In the case of the PSC power plant, there were also photovoltaic panels, comprising 48% of the share in mass. The mass percentage share of the remaining construction elements was similar for both power plants. In the case of the supporting structure, its share was about 17% for sc-Si and about 16% for PSC (mostly made of steel), for the inverter station it was about 13% for sc-Si and about 14% for PSC (elements made mainly of steel (about 42%) and aluminum (about 38%)), while for the electrical installation it was about 2% in both cases (it is mostly made of copper) (Figure 6 and Figure 7) (data obtained from the investor and manufacturers).

The energy sector is undergoing changes, and the gradual transition to renewable energy sources is more than obvious. Not every technical object that potentially seems sustainable remains so after its life cycle. This topic is one of the most common problems with photovoltaic panels. They are largely recyclable. Materials such as glass, aluminum and semiconductors can be recovered and then reused. So far, the most common recycling methods have been based on mechanical, thermal and chemical processes. Recycling photovoltaic panels involves dismantling the frame located on the PV modules to separate the aluminum and glass parts. Next, the cables and the junction box are removed. The prepared material is then cut and crushed. Almost all glass from PV modules (approx. 95%) can be reused. The same applies to the recycling of external metal parts. Photovoltaic cells are also recycled (approx. 80–90% can be reused). The remaining elements are subjected to high-temperature thermal treatment (up to 500 °C). In this process, the polymer materials surrounding the individual parts of the PV panels evaporate. In this way, the silicon cells become ready for further processing. Their recycling involves acid etching of the silicon particles and enriching the structure so that their original properties are restored. Therefore, the common belief that photovoltaic panels are not suitable for recycling is a myth. However, this is a process that requires time for wide implementation and further research to achieve the full potential of proper recycling of all PV panel components. For this reason, close cooperation between design and recycling units is necessary to ensure the ability to sustainably manage post-consumer waste through conscious eco-design. Although in most cases PV panels can be recycled and, thus, their negative impact on the environment can be minimized, one must be aware of the risk of improper post-consumer management. Landfilling waste poses a huge threat to the natural environment. Considering the heavy metals present in photovoltaic modules (e.g., lead and tin), this can cause serious pollution problems. In addition, they also contain valuable metals such as silver and copper, which further supports their recycling [54,55].

### 2.4. Life Cycle Impact Assessment (LCIA)

**Figure 8:**
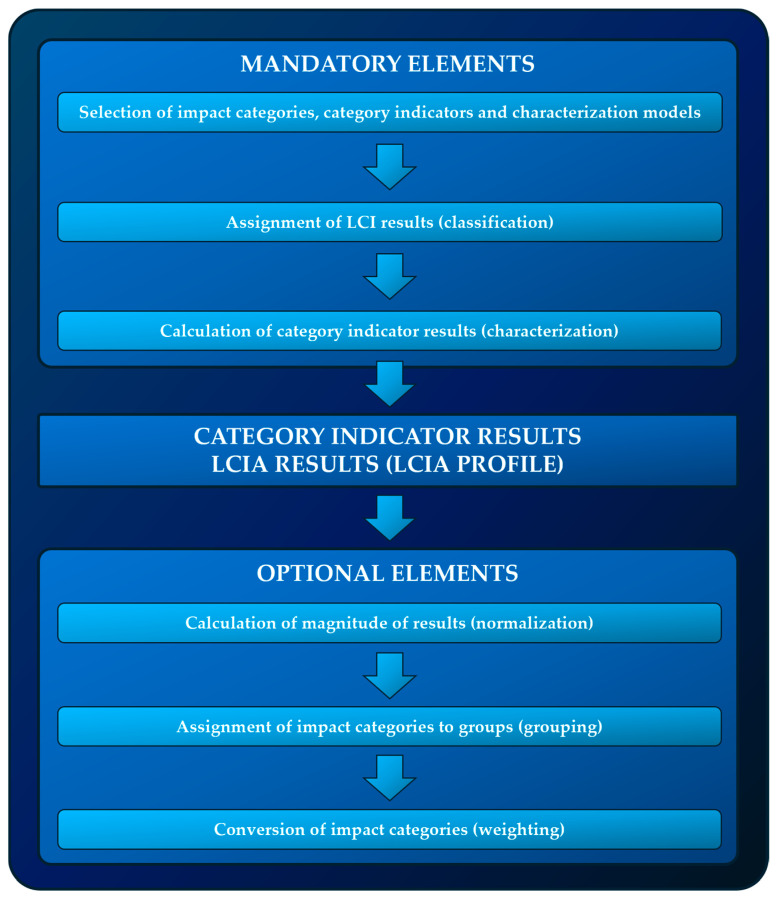
LCIA stages of a photovoltaic power plant.

The third stage of LCA studies consists of determining the potential impact of the studied object on the environment. For this reason, the key element of LCIA is to characterize and then classify emissions resulting from the life cycle of the photovoltaic power plant, and then to link these emissions to the effects they may cause to the environment. The obtained results are presented in the form of category indicators (quantitative presentation of impact categories). The LCIA phase consists of mandatory and optional elements. The mandatory ones include the selection of impact categories, category indicators, characterization models, classification and characterization. The optional elements include normalization, grouping and weighting (Figure 8). All mandatory and optional elements were used in the assessment. The analyses were performed using SimaPro 9.5 (Ecoinvent 3.9.1) software, and the IMPACT World+, IPCC and CED models [56,57,58,59].

Classification involves assigning LCI results to impact categories. Specialized analytical software is used for this purpose (in the case of this study, it was SimaPro 9.5) [60,61].

Characterization and conversion of LCI results into category indicator results are very complex processes. They consist of converting LCI results using specific characterization parameters and then presenting them in the form of relative shares in each category. The models used at this stage were IMPACT World+, IPCC and CED [62,63,64].

Normalization involves determining the size of the impact category indicator results in relation to reference information. It allows for determining the relative weight of the indicator in each geographical horizon (for example Europe) or in relation to a person (for example an average European resident) in an adopted time horizon. As part of the conducted assessment, the normalization procedure was used to prepare the LCIA results for the next step—grouping and weighting [65,66].

Grouping and weighting is a process in which a weighting factor is determined and assigned to impact categories, and then multiplied by normalized index values. In the conducted research, weighting was performed only on complete, internationally recognized sets of coefficients, developed for all considered categories. The implementation of this stage allowed results to be obtained in units, which could be compared (both within the impact category, as well as the impact areas and the final environmental indicator). Analyses of the characterization, normalization, grouping and weighting were performed using SimaPro 9.5 software [67,68,69].

**Figure 9:**
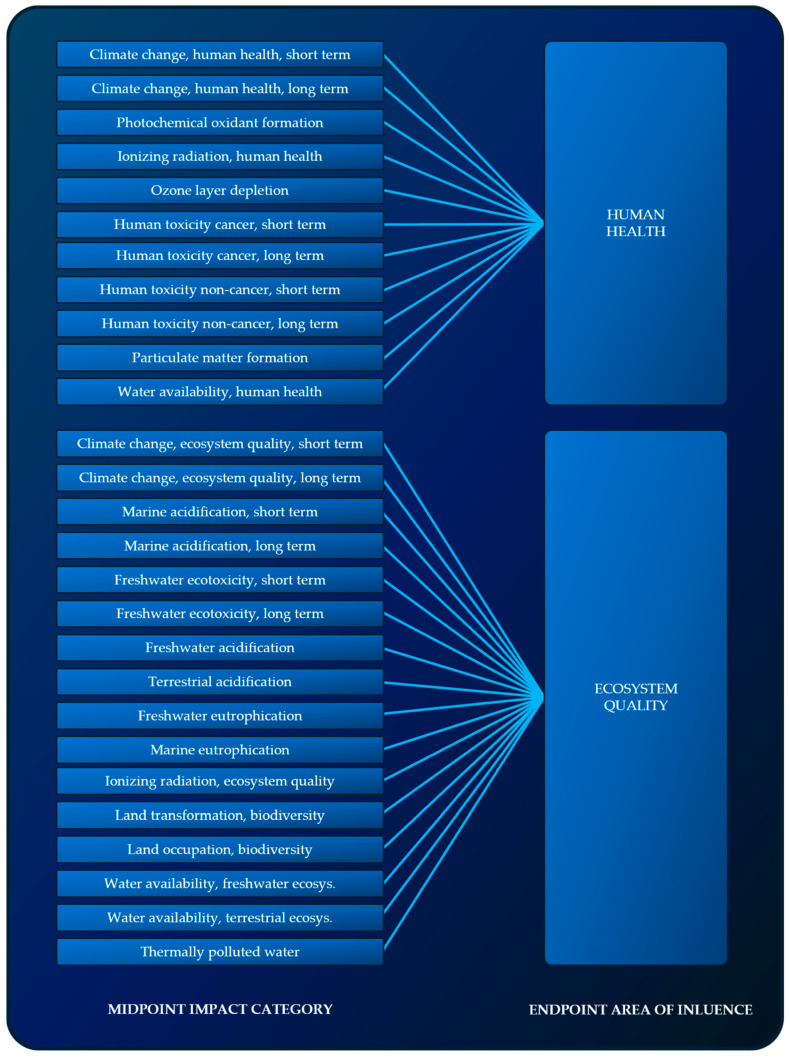
Impact categories and impact areas analyzed within the IMPACT World+ method.

The IMPACT World+ method is the latest update of the IMPACT 2002+, LUCAS and EDIP methods. It allows for the determination of environmental impacts within 27 impact categories, which can be grouped into two impact areas (impacts on ecosystem quality and human health) and an environmental final indicator. IMPACT World+ includes the broadest set of midpoint impact categories compared to other methods used in LCA analyses. Impact categories are further divided into those covering short-term damages (up to 100 years after emission) and long-term damages (occurring even after 100 years after emission) (Figure 9) [70,71,72].

The IPCC 2021 GWP model was also used in the analyses. It allowed the greenhouse potential (GWP) to be determined. It was developed by the Intergovernmental Panel on Climate Change. Carbon dioxide IV (CO_2_) is the compound in this model to which the greenhouse potential is referred. For this reason, the results are presented in kg CO_2_ eq. The study assumed a 100-year period of GHG impact on the greenhouse effect. The analysis of the impact on the environment included three impact categories: GWP100—fossil, biogenic and land transformation [73,74,75,76].

The last method used for the analyses was cumulative energy demand (CED). It allows for determining how much primary energy was used in the life cycle of the research object. The results are presented in MJ or MWh. As part of the assessment, the results were analyzed for all available impact categories—three covering non-renewable sources (fossil, nuclear and biomass) and three covering renewable sources (biomass, wind, solar, geothermal and water) [77,78,79].

### 2.5. Interpretation

Interpretation is not only the last stage of LCA; it is present in each of the earlier stages of the conducted procedure. Its aim is to analyze the results and verify them in terms of the previously formulated goal and scope of the research. The completeness of the analysis within the conducted research was checked with a positive result. The data necessary for the interpretation were completed. A compliance check was also performed. The methods used, the assumptions adopted, the detail and depth of the analysis and the accuracy of the data used were consistent with the goal and scope of the research. The obtained assessment results and their interpretation are presented in Section 3 and Section 4 [80,81,82].

## 3. Results

The obtained results of the analyses were divided into three main sections covering the analytical models used: IMPACT World+ Endpoint version 1.02 (Section 3.1), IPCC 2021 GWP 100 version 1.01 (Section 3.2) and CED—Cumulative Energy Demand version 1.11 (Section 3.3). The Ecoinvent 3.9.1 database was used for the research. The data used were obtained directly from manufacturers and companies involved in the operational and post-consumer management of the studied photovoltaic power plants; when this was not possible, the data were obtained from SimaPro software databases. Within the IMPACT World+ model, the results were grouped into three areas: impact categories (covering 27 categories, Section 3.1.1), areas of influence (covering 2 areas, Section 3.1.2) and total impact (presenting the total impact on the environment, Section 3.1.3).

### 3.1. IMPACT World+

The presented research results using the IMPACT World+ model were expressed in three types of units: DALY, PDF × m^2^ × yr, and EUR. DALY (disability-adjusted life years) is an indicator used to determine health status. It expresses the total number of years of life lost due to premature death or damage to health (because of injury or disease). It was developed by WHO. One DALY means the loss of one year of health, which may be caused by disability or death. The PDF × m^2^ × yr unit describes the level of biodiversity. PDF means the potentially disappeared fraction of species, which is calculated on a given area (hence, m^2^) or in each volume (hence, m^3^), in each period (hence, the addition of years). EUR (euro, €) is the currency of the European Union.

#### 3.1.1. Impact Categories

Among the impact categories characterized by the highest potential negative impact on human health in the life cycle of a photovoltaic power plant based on monocrystalline (sc-Si) modules, we can distinguish water availability, human health and climate change, long term. In both cases, photovoltaic panels were the elements of the power plant with the highest level of harmful impact; in the case of the categories first mentioned, it was 2.13 × 10^0^ DALY (landfill) and 1.27 × 10^0^ DALY (recycling), while for the second, it is was −2.47 × 10^−2^ DALY (landfill) and 1.82 × 10^−2^ DALY (recycling). In the scope of impact categories related to ecosystem quality, the most potential harmful impacts were recorded for freshwater ecotoxicity, long term, climate change and ecosystem quality, long term. Also in these areas, photovoltaic panels were characterized by the highest values of destructive impacts. For the first category, they had the values of 1.58 × 10^−2^ PDF × m^2^ × yr (landfill) and 6.78 × 10^−4^ PDF × m^2^ × yr (recycling), while for the second, they had the values of 6.62 × 10^−5^ PDF × m^2^ × yr (landfill) and 4.56 × 10^−5^ PDF × m^2^ × yr (recycling). The main reason for such a high level of adverse impact on the environment is the very energy-intensive process of manufacturing photovoltaic cells from monocrystalline silicon, using the Czochralski method. It also produces a large amount of post-production waste during the cutting of the cells, from a round shape to one close to a rectangular shape. In the case of the remaining elements of the examined photovoltaic power plant (support structure, inverter station, electrical installation and transformer), the impact categories characterized by the maximum value of negative impact on human health included water availability, human health, human toxicity and non-cancer, long term. In the area of impact on ecosystem quality, these are the categories of freshwater ecotoxicity, long term, and climate change, ecosystem quality, long term (analogous to photovoltaic panels). The electrical installation was distinguished by the lowest total level of harmful impact on the environment (both on human health and ecosystem quality). This was due to the lowest mass of materials necessary for its production in comparison to the other groups of elements (approx. 2% of the total mass of the power plant—Figure 7). However, electrical wires (which constitute the largest share in its structure) are made primarily of copper (for which the processes of ore extraction and subsequent production are very energy-intensive) and polymer materials constituting insulation (recycling of which is a complex and technologically demanding process) (Table 1).

The second analyzed photovoltaic power plant was a facility based on perovskite modules (PSC). And, in this case, the highest level of adverse impact, both in terms of impact on human health and on the quality of the ecosystem, was characterized by the life cycle of the photovoltaic panels. The impact categories distinguished by the maximum value of potential harmful impact were again: water availability, human health (1.92 × 10^0^ DALY for landfill, and 1.15 × 10^0^ DALY for recycling), climate change, human health, long term (2.23 × 10^−2^ DALY for landfill, and 1.64 × 10^−2^ DALY for recycling), freshwater ecotoxicity, long term (1.43 × 10^−3^ PDF × m^2^ × yr for landfill, and 6.14 × 10^−4^ PDF × m^2^ × yr for recycling), and climate change, ecosystem quality, long term (5.99 × 10^−5^ PDF × m^2^ × yr for landfill, and 4.12 × 10^−5^ PDF × m^2^ × yr for recycling). Photovoltaic panels have the largest share in the total mass of the power plant (approx. 48%—Figure 7). However, their production is less energy- and material-intensive compared to monocrystalline silicon panels; hence, lower values of destructive impact were noted in the above-mentioned impact categories. In relation to other construction elements (support structures, inverter stations, electrical installations, and transformers), the impact categories with the highest degree of dangerous impact on health again include water availability, human health and human toxicity non-cancer, long-term. In turn, in terms of impact on the ecosystem, these are again the categories of freshwater ecotoxicity, long term, and climate change, ecosystem quality, long term (as in the case of sc-Si panels). Similarly, due to the lowest percentage share in the power plant mass (approx. 2%—Figure 7), the electrical installation elements were characterized by the lowest degree of adverse impact on the environment (both in terms of impact on human health and the quality of the ecosystem) (Table 2).

According to the above, in the perspective of the life cycle of materials and elements of both analyzed photovoltaic power plants, the categories of impact with the highest level of harmful impact on human health include water availability, human health, climate change and human health, long term. In turn, in terms of impact on ecosystem quality, these are the categories of freshwater ecotoxicity, long term, and climate change, ecosystem quality, long term. For this reason, a decision was made to conduct a more detailed analysis of destructive impacts in these areas.

**Figure 10:**
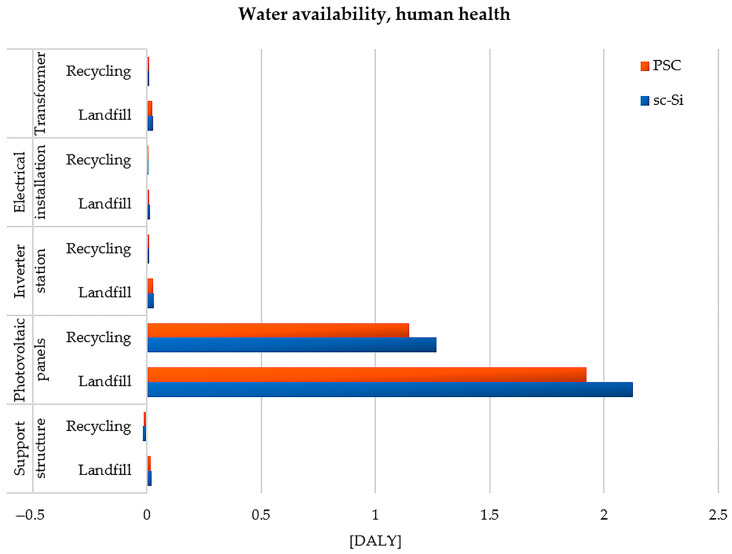
Characterization of environmental consequences of processes affecting water availability, affecting human health, in the life cycle of the studied photovoltaic power plants (model IMPACT World+ Endpoint ver. 1.02, unit: DALY).

The first of the categories considered were water availability and human health. It illustrates how the life cycle of the tested materials and construction elements affects the availability of water, the deficiency of which can directly affect health (especially in areas with a lower level of economic development and those where access to water is very limited). The maximum value of the negative impact in this area characterizes the life cycle of monocrystalline photovoltaic panels (sc-Si), which would be stored in a landfill after the end of their use (2.13 × 10^0^ DALY). The use of recycling processes would significantly reduce the amount of hazardous impact on health (less about 8.58 × 10^−1^ DALY). For all the assessed elements, the life cycle of a power plant based on perovskite cells (PSC) generates fewer adverse effects on water availability, compared to a power plant based on monocrystalline silicon cells (sc-Si). Among the processes related to the reduction in water availability in the life cycles of both technical facilities examined, we can distinguish primarily the consumption of water from various sources (lakes, rivers, wells, etc.) and its use for cooling turbines (during electricity generation). The issue of limited water availability is one of the key environmental, economic and health problems. People overuse available water resources and drain huge areas of land (e.g., for the purpose of building mines). Nowadays, its consumption is greater than the increase, which leads to the depletion of resources. Another problem is the reduction in water quality through its contamination caused by, for example, agriculture, industry, transport, low emissions, detergents or poorly secured or illegal landfills. The average water consumption in European countries is approx. 1200 m^3^/yr, in USA—aprox. 2500 m^3^/yr, and in Ethiopia—aprox. 360 m^3^/yr (due to difficult accessibility). Almost 900 million people in the world do not have access to water considered suitable for drinking. In developing countries, lack of access to clean water is the cause of about 80% of diseases. Therefore, special attention should be paid to the issue of water demand in all industrial processes, including those in the life cycle of photovoltaic power plants (from the extraction of raw materials to the post-consumer management of materials and construction elements) (Figure 10) [83,84,85].

**Figure 11:**
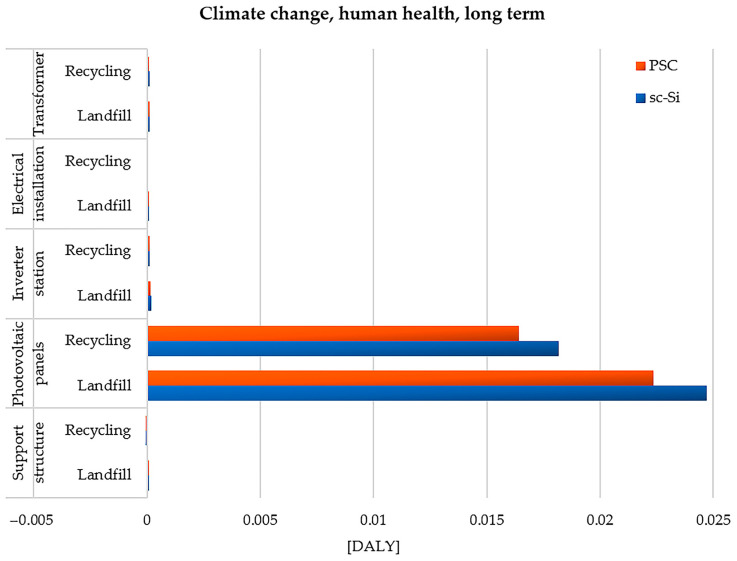
Characterization of the environmental consequences of emissions of climate-changing substances affecting human health (long-term perspective) over the life cycle of the studied photovoltaic power plants (model IMPACT World+ Endpoint ver. 1.02, unit: DALY).

The second analyzed impact category is climate change, human health, long term. It includes emissions of chemical compounds throughout the entire life cycle of photovoltaic power plants, which contribute to the deepening of climate change and, consequently, are characterized by a harmful effect on health. The long-term perspective considers the potential impact of the assessed substances on the environment for a period exceeding 100 years from the moment of emission. In this case, the greatest degree of dangerous impact is also distinguished by the life cycle of monocrystalline photovoltaic panels, which would be placed in a landfill (2.47 × 10^−2^ DALY). However, recycling would, to some extent, make it possible to reduce the level of adverse impact on human health in the area under consideration (less about 6.54 × 10^−3^ DALY). The life cycle of the sc-Si power plant causes more harmful impacts in terms of climate change than the life cycle of the PSC power plant. Among the chemical compounds influencing climate change, in the life cycles of both studied power plants, the highest values of destructive impact were characterized by emissions: carbon dioxide, methane, dinitrogen monoxide, sulfur hexafluoride, tetrafluoromethane (CFC-14), hexafluoroethane (HFC-116), and trifluoromethane (HFC-23). Carbon dioxide is an important part of the carbon cycle in nature, because it is a product of combustion and respiration. Large amounts of it are emitted in the processes of manufacturing materials and components of the tested power plants, which are characterized by a high level of energy and material intensity. The increase in its concentration in the environment results in a deepening of the greenhouse effect and many other dangerous consequences for human health and the quality of the ecosystem. On the other hand, life on Earth would not be possible without CO_2_. The problem is not the existence of carbon dioxide itself, but the large increase in its concentration, which is currently occurring at an increasingly rapid pace. (Figure 11) [86,87].

**Figure 12:**
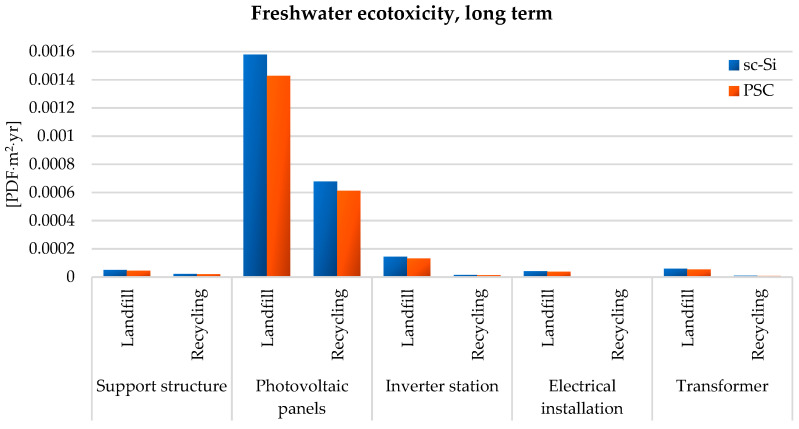
Characterization of the environmental consequences of the emission of substances with ecotoxic effects on freshwater ecosystems (long-term perspective) in the life cycle of the studied photovoltaic power plants (model IMPACT World+ Endpoint ver. 1.02, unit: PDF × m^2^ × yr).

Among the impact categories characterizing the impact values on the quality of ecosystems, the issue of access to water suitable for consumption is also extremely important. Within the impact category of freshwater ecotoxicity, long term, the maximum degree of negative impact was characterized by the life cycle of monocrystalline photovoltaic panels (sc-Si), which would not be recycled after the end of their use, but would be placed in a landfill (1.58 × 10^−3^ PDF × m^2^ × yr). Post-consumer management in the form of recycling would significantly reduce the level of unsafe impact on the environment (less about 9.00 × 10^−4^ PDF × m^2^ × yr). For each of the analyzed structural elements of both power plants, the use of recycling processes would result in a significant reduction in the value of adverse impacts on the environment. The life cycle of the power plant based on photovoltaic modules made of perovskites is characterized by a lower level of harmful impact in the analyzed area compared to the sc-Si power plant. Among the structural materials of the assessed power plants, the life cycle of which has the greatest impact on the increase in the ecotoxicity of freshwater, the following can be distinguished: copper, aluminum, iron, strontium, nickel, zinc, manganese, cadmium, and vanadium. Copper is an element that participates in several biochemical reactions. It plays an important role in photosynthesis and in the formation of proteins. However, the exploitation and processing of copper ore deposits (and other metals) results in the introduction of many harmful substances into the environment (e.g., through the discharge of mine waters, sewage from smelters and enrichment plants, dust emissions from smelters, the formation of smelter and flotation waste, etc.). The largest number of harmful emissions goes to the aquatic environment (landfill leachates, soil runoff, sewage discharge, etc.). For this reason, it is extremely important to increase the level of recycling of copper waste. Its recycling is characterized by a high level of efficiency and energy saving. It requires about 85% less energy input compared to primary production. Copper can be recovered many times, because its processing does not deteriorate the quality and functional properties. Due to the high energy consumption of the processes related to the production of copper elements and the huge interference in the environment of its mining and processing processes, despite the fact that the percentage share of copper in the mass of the studied power plants is small (about 2% for sc-Si and about 1% for PSC—Figure 6), its life cycle has a large impact on the pollution of the aquatic environment (Figure 12) [88,89,90].

**Figure 13:**
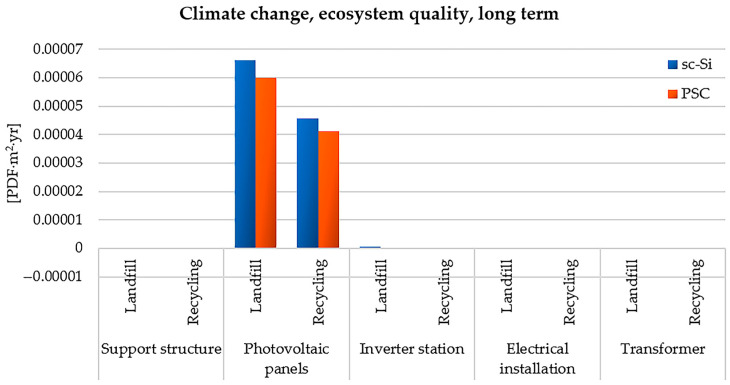
Characterization of the environmental consequences of emissions of substances causing climate change, affecting the quality of ecosystems (long-term perspective), in the life cycle of the studied photovoltaic power plants (model IMPACT World+ Endpoint ver. 1.02, unit: PDF × m^2^ × yr).

The last of the analyzed impact categories is climate change, ecosystem quality, long term. It is therefore clear that the issue of climate change is crucial not only in the area of impact on human health, but also on the quality of the ecosystem. As in the case of the previously characterized categories, the maximum value of destructive impact was distinguished by the life cycle of photovoltaic panels of the sc-Si power plant, stored after the end of operation (6.62 × 10^−5^ PDF × m^2^ × yr). Recycling would significantly reduce the negative impact in the assessed area (less about 2.07 × 10^−5^ PDF × m^2^ × yr). The life cycle of a photovoltaic power plant based on monocrystalline silicon panels generates more hazardous environmental consequences compared to the life cycle of a power plant based on perovskite panels. The chemical compounds occurring in the life cycle of the considered power plants that cause the most adverse impacts in the area of climate change includes: methane, carbon dioxide, dinitrogen monoxide, sulfur hexafluoride, tetrafluoromethane (CFC-14), hexafluoroethane (HFC-116), and trifluoromethane (HFC-23). Methane is a greenhouse gas that poses a significant threat to the quality of ecosystems and human health. During the first 20 years of its presence in the atmosphere, its impact on the climate is about 85 times greater than the same mass of carbon dioxide. Its largest sources are agriculture and the energy industry (using conventional energy sources). Reducing its emissions is possible, among other things, by moving away from fossil fuels and securing abandoned oil and gas wells and closed mines against leaks. Processes related to the production and management of materials and components of photovoltaic power plants, requiring large inputs of energy and matter, are characterized by a particularly high level of methane emissions to the environment (Figure 13) [91,92].

#### 3.1.2. Areas of Influence

**Figure 14:**
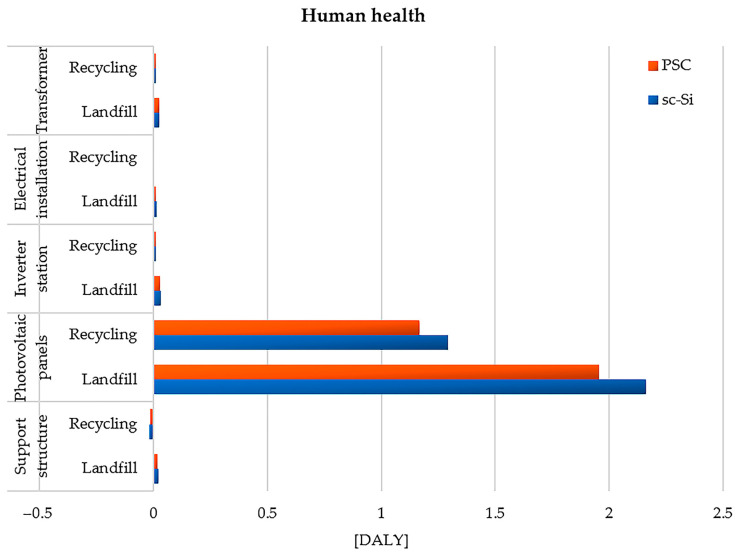
Characterization of the environmental consequences for human health during the life cycle of the studied photovoltaic power plants (model IMPACT World+ Endpoint ver. 1.02, unit: DALY).

As part of the analyses, the impact of the life cycles of the studied power plants on two areas of impact was also assessed—human health and the quality of the ecosystem. The highest level of the total adverse impact on human health was characteristic of the life cycle of photovoltaic panels (sc-Si: 2.16 × 10^0^ DALY for landfill, and 1.29 × 10^0^ DALY for recycling, PSC: 1.95 × 10^0^ DALY for landfill, and 1.17 × 10^0^ DALY for recycling). The remaining elements of the power plant were characterized by significantly lower values of the total harmful impact: inverter station (sc-Si: 3.21 × 10^−2^ DALY for landfill, and 1.09 × 10^−2^ DALY for recycling, PSC: 2.91 × 10^−2^ DALY for landfill, and 9.84 × 10^−3^ DALY for recycling), transformer (sc-Si: 2.26 × 10^−2^ DALY for landfill, and 1.05 × 10^−2^ DALY for recycling, PSC: 2.37 × 10^−2^ DALY for landfill, and 9.49 × 10^−3^ DALY for recycling), support structure (sc-Si: 1.96 × 10^−2^ DALY for landfill, and −1.70 × 10^−2^ DALY for recycling, PSC: 1.77 × 10^−2^ DALY for landfill, and −1.54 × 10^−2^ DALY for recycling), and electrical installation (sc-Si: 1.19 × 10^−2^ DALY for landfill, and 2.38 × 10^−3^ DALY for recycling, PSC: 1.08 × 10^−2^ DALY for landfill, and 2.16 × 10^−3^ DALY for recycling). For each element considered, for both power plants studied, the life cycle with post-consumer disposal in the form of storage had more negative consequences for human health than the cycle with the use of recycling processes. The power plant based on monocrystalline silicon (sc-Si) technology caused more destructive impacts in the assessed area compared to the perovskite (PSC) power plant (Figure 14 and Table 3).

**Figure 15:**
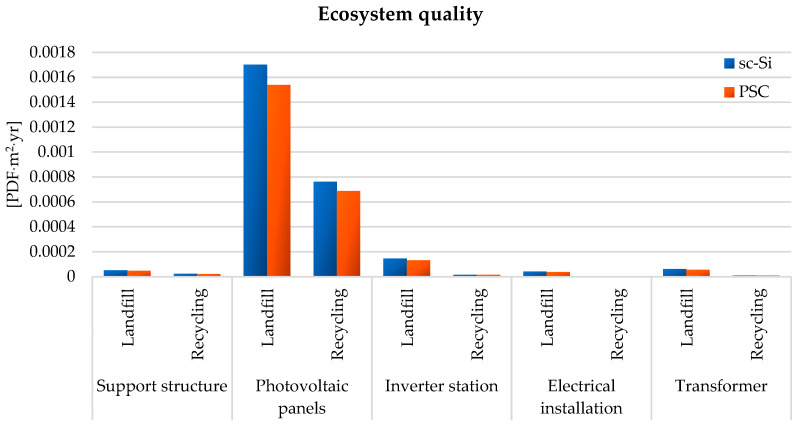
Characterization of environmental consequences for the quality of the ecosystem during the life cycle of the studied photovoltaic power plants (model IMPACT World+ Endpoint ver. 1.02, unit: PDF × m^2^ × yr).

A similar situation occurred in the case of the impact area covering the quality of the ecosystem—the maximum value of the total harmful impact was noted for the life cycle of photovoltaic panels (sc-Si: 1.70 × 10^−3^ PDF × m^2^ × yr for landfill and 7.62 × 10^−4^ PDF × m^2^ × yr for recycling; PSC: 1.54 × 10^−3^, PDF × m^2^ × yr for landfill and 6.89 × 10^−4^ PDF × m^2^ × yr for recycling). Other elements were characterized by a much lower degree of combined destructive impact: inverter station (sc-Si: 1.47 × 10^−4^ PDF × m^2^ × yr for landfill and 1.63 × 10^−5^ PDF × m^2^ × yr for recycling; PSC: 1.33 × 10^−4^, PDF × m^2^ × yr for landfill and 1.47 × 10^−5^ PDF × m^2^ × yr for recycling), transformer (sc-Si: 6.08 × 10^−5^ PDF × m^2^ × yr for landfill and 9.79 × 10^−6^ PDF × m^2^ × yr for recycling; PSC: 5.50 × 10^−5^, PDF × m^2^ × yr for landfill and 8.86 × 10^−6^ PDF × m^2^ × yr for recycling), support structure (sc-Si: 5.18 × 10^−5^ PDF × m^2^ × yr for landfill and 2.32 × 10^−5^ PDF × m^2^ × yr for recycling; PSC: 4.68 × 10^−5^ PDF × m^2^ × yr for landfill and 2.10 × 10^−5^ PDF × m^2^ × yr for recycling), and electrical installation (sc-Si: 4.22 × 10^−5^ PDF × m^2^ × yr for landfill and 1.35 × 10^−6^ PDF × m^2^ × yr for recycling; PSC: 3.81 × 10^−5^ PDF × m^2^ × yr for landfill and 1.22 × 10^−6^ PDF × m^2^ × yr for recycling). Again, each element of both power plants had a life cycle with post-consumer management in the form of recycling causing fewer adverse effects on ecosystem quality compared to the life cycle involving landfill. The PSC power plant had fewer negative impacts in the studied range than the sc-Si power plant (Figure 15 and Table 3).

The impact areas were also analyzed in terms of potential environmental costs that the life cycles of the studied photovoltaic power plants entail (in the EUR unit—euro currency, €). The life cycle of a power plant based on monocrystalline photovoltaic panels (sc-Si) is associated with higher environmental costs incurred as a result of deterioration of human health (total: 7.93 × 10^6^ EUR for landfill and 4.25 × 10^6^ EUR for recycling) and lowering the quality of the ecosystem (total: 4.36 × 10^6^ EUR for landfill and 2.27 × 10^6^ EUR for recycling), compared to the life cycle of a power plant based on perovskite panels (PSC). In principle, both post-consumer management processes in the form of storage and recycling cause higher environmental costs in the area of human health than in the quality of the ecosystem (Table 4).

#### 3.1.3. Total Impact

**Figure 16:**
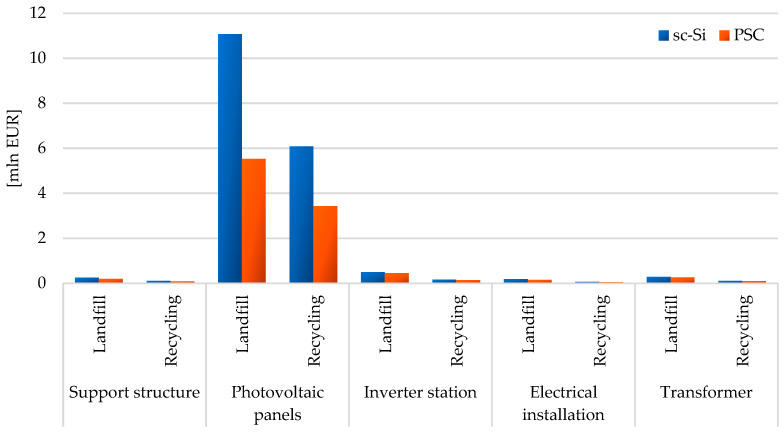
Grouping and weighing environmental consequences in the life cycle of the studied photovoltaic power plants, taking into account different post-consumer management scenarios (model IMPACT World+ Endpoint ver. 1.02, unit: mln EUR).

The element of both assessed photovoltaic power plants characterized by the highest environmental cost of the life cycle are photovoltaic panels; especially if their form of post-consumer management is landfilling (sc-Si: 1.11 × 10^7^ EUR, PSC: 5.53 × 10^6^ EUR). The use of recycling processes would significantly reduce the environmental cost of their life cycle (sc-Si: o 4.98 × 10^6^ EUR, PSC: o 2.09 × 10^6^ EUR). In the case of all other elements (support structure, inverter station, electrical installation, transformer), the environmental cost of their life cycle is lower for a photovoltaic power plant based on perovskite cell technology and for the form of post-consumer management using recycling processes. The lowest environmental cost is distinguished by the life cycle of an electrical installation, which will be recycled after the end of use (sc-Si: o 1.85 × 10^5^ EUR, PSC: o 1.47 × 10^5^ EUR). As mentioned earlier, this is due to the small mass of this element compared to the total mass of the power plant (Figure 16).

**Figure 17:**
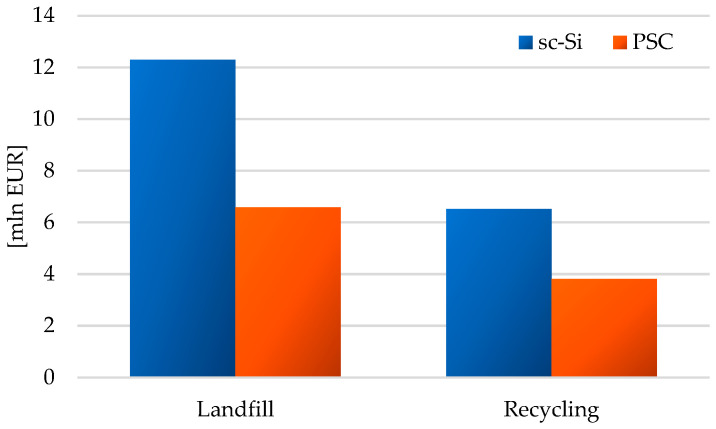
Grouping and weighing the environmental consequences for the ecosystem quality in the life cycle of the studied photovoltaic power plants (model IMPACT World+ Endpoint ver. 1.02, unit: mln EUR).

Total environmental life cycle cost of a photovoltaic power plant using monocrystalline silicon photovoltaic panels (landfill: 1.23 × 10^7^ EUR, recycling: 6.52 × 10^6^ EUR) was higher compared to the perovskite power plant (landfill: 6.59 × 10^6^ EUR, recycling: 3.81 × 10^6^ EUR). This was determined by the size of the environmental cost of photovoltaic panels, which in the case of sc-Si power plants required much higher energy and material inputs compared to those used in PSC power plants. The form of post-consumer management also had a key impact on the total value of the environmental cost of the life cycle of a photovoltaic power plant (Figure 17).

### 3.2. IPCC 2021

Thanks to the application of the second analytical model—IPCC 2021 GWP100 version 1.01—it was possible to determine the size of the environmental consequences for greenhouse gas emissions in the life cycle of the assessed photovoltaic power plants. The largest GHG emissions in the life cycles of both power plants occurred as a result of the use of fossil sources (fuels and mineral raw materials, impact category: GWP100—fossil). As a result, there were emissions of, among others, carbon dioxide, methane, dinitrogen monoxide, sulfur hexafluoride, tetrafluoromethane (CFC-14), trifluoromethane (HFC-23), hexafluoroethane (HFC-116), dichlorodifluoromethane (CFC-12). Their highest level was recorded in the life cycle of monocrystalline silicon photovoltaic panels (landfill: 1.01 × 10^6^ kg CO_2_ eq; recycling: 5.51 × 10^5^ kg CO_2_ eq). As mentioned earlier, the production of cells using the Czochralski method is an extremely energy-intensive process, which as a consequence generates many substances with a destructive effect on the environment (Table 5).

**Figure 18:**
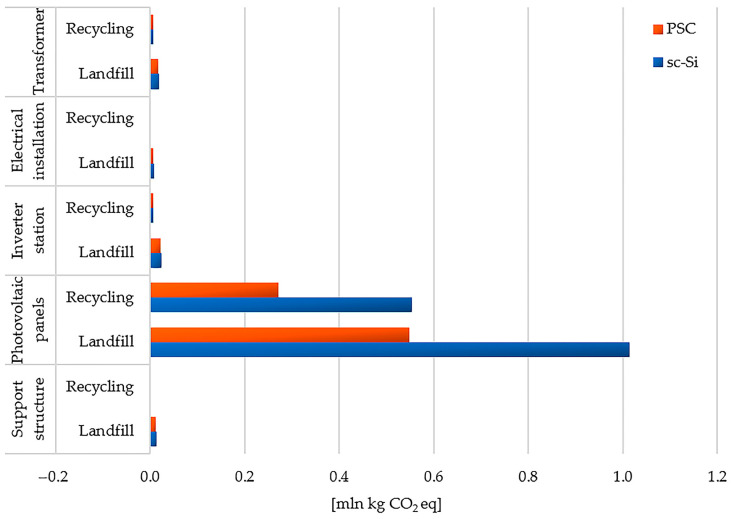
Characterization of environmental consequences for greenhouse gas emissions in the life cycle of the studied photovoltaic power plants, taking into account different post-consumer management scenarios (model IPCC 2021 GWP100 ver. 1.01, unit: mln kg CO_2_ eq).

The highest total level of greenhouse gas emissions in the life cycles of both studied power plants was distinguished by photovoltaic panels (sc-Si: landfill −1.01 × 10^6^ kg CO_2_ eq, recycling −5.54 × 10^5^ kg CO_2_ eq; PSC: landfill −5.49 × 10^5^ kg CO_2_ eq, recycling −2.71 × 10^5^ kg CO_2_ eq). For all elements of the analyzed technical facilities, the use of recycling processes is associated with lower GHG emissions compared to landfill management. The life cycles of PSC power plant elements generate less greenhouse gasses than SC power plants (Figure 18).

**Figure 19:**
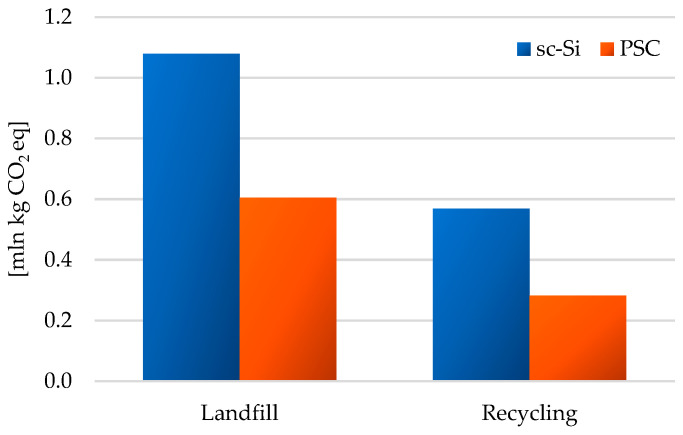
Characterization of environmental consequences for greenhouse gas emissions in the life cycle of the studied photovoltaic power plants (model IPCC 2021 GWP100 ver. 1.01, unit: mln kg CO_2_ eq).

The total level of greenhouse gas emissions was higher for the management of materials and components of both photovoltaic power plants in the form of landfill (sc-Si: 1.08 × 10^6^ kg CO_2_ eq; PSC: 5.69 × 10^5^ kg CO_2_ eq) compared to recycling management (sc-Si: 6.05 × 10^5^ kg CO_2_ eq; PSC: 2.82 × 10^5^ kg CO_2_ eq). The life cycle of a power plant based on monocrystalline silicon panels was distinguished by a higher level of GHG emissions than the life cycle of a power plant based on perovskite panels (Figure 19).

### 3.3. Cumulative Energy Demand (CED)

The last model used in the study: CED—Cumulative Energy Demand version 1.11, allowed for determining the energy demand in the life cycle of the analyzed photovoltaic power plants. Due to the structure of the energy mix in Europe, the largest amount of energy in the life cycles of both analyzed power plants came from non-renewable sources (impact category: non-renewable fossil), mainly from crude oil, coal and gas. Among renewable sources, waterpower stood out with the highest degree of coverage of the energy demand of the research objects (impact category: renewable water). The highest level of energy demand from both non-renewable sources (total: 1.46 × 10^7^ MJ), and renewable (total: 5.87 × 10^6^ MJ) was noted for the life cycle of monocrystalline silicon panels, which after the end of their use were placed in a landfill. The increasingly rapid consumption of non-renewable energy sources is not only associated with the depletion of their resources; their exploitation also results in a decrease in the quality of ecosystems and the degradation of the environment. The most negative environmental consequences, and consequently, the environmental costs, are caused by opencast mining. Hence, the introduction of changes in the energy mixes of countries, toward increasing the share of renewable energy sources, is such an important problem today (Table 6).

**Figure 20:**
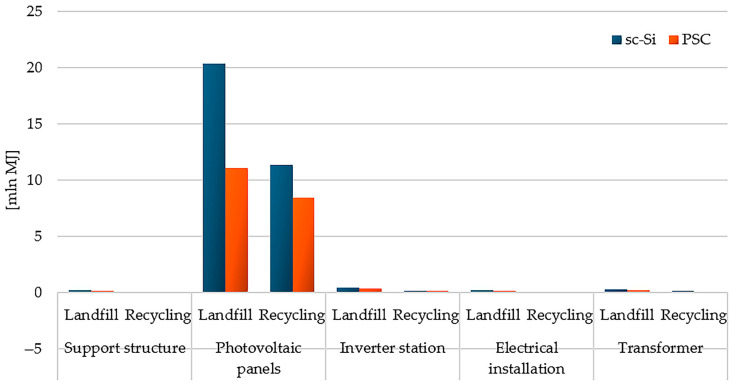
Characterization of energy demand in the life cycle of the studied photovoltaic power plants, taking into account different post-consumer development scenarios (model CED—Cumulative Energy Demand ver. 1.11, unit: mln MJ).

The high mass share of photovoltaic panels in the masses of the tested power plants (sc-Si—approx. 53%; PSC—approx. 48%, Figure 7) and the high demand for matter and energy in their life cycles make them the elements of the power plant with the highest cumulative demand for energy. In turn, the lowest demand in this respect, due to its small mass, was characteristic of the electrical installation with recycling as a form of post-consumer management (total: sc-Si −6.03 × 10^4^ MJ; PSC −4.57 × 10^4^ MJ). In the case of each of the analyzed power plant components, post-consumer management through landfilling was a source of greater energy demand in the life cycle than recycling processes, in which this energy could have been recovered (in a broader perspective) by using other materials and components in production (high energy costs of primary production are avoided) (Figure 20).

**Figure 21:**
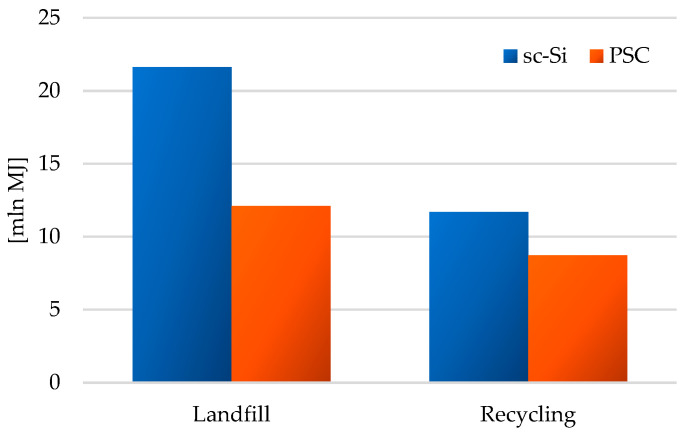
Characterization of energy demand in the life cycle of the studied photovoltaic power plants (model CED—Cumulative Energy Demand ver. 1.11, unit: mln MJ).

The life cycle of a photovoltaic power plant based on sc-Si technology is characterized by a higher total cumulative energy demand (landfill: 2.16 × 10^7^ MJ; recycling: 1.172.16 × 10^7^ MJ) compared to a power plant based on PSC technology (landfill: 1.212.16 × 10^7^ MJ; recycling: 8.732.16 × 10^6^ MJ). The use of recycling processes allows for a significant reduction in energy demand in the life cycles of both tested technical objects (Figure 21).

### 3.4. Comparison of the Total Impact on the Environment

Table 7 presents the results of the total environmental impact of the life cycle of a photovoltaic power plant based on monocrystalline silicon (sc-Si) and perovskite (PSC) modules for two post-consumer development scenarios (landfill and recycling). In terms of impact on human health, environmental quality, greenhouse gas emissions and total energy demand, the highest level of negative impact on the environment was observed in the life cycle of a monocrystalline power plant (sc-Si), which materials, components and components would be disposed of in a landfill after the end of their service life, while the lowest impact was observed in the case of a perovskite power plant (PSC), for which the post-consumer development scenario would include recycling processes. Therefore, perovskites can be considered as a construction material that better fits into the main assumptions of sustainable development, compared to the currently most commonly used monocrystalline silicon.

## 4. Summary and Conclusions

The increase in the standard of living and the number of people in the world means that the global demand for energy is increasing year by year. Currently, most energy is obtained from fossil sources, the exploitation of which is characterized by a huge, destructive impact on the environment. The production, exploitation and post-consumer management of renewable sources also involve some use of non-renewable resources, but to a much smaller extent that that of their conventional counterparts. Photovoltaic power plants are considered “environmentally friendly”, but the technologies used in their life cycles may fit into the main assumptions of sustainable development [93,94,95].

The main objective of the study was achieved by assessing the environmental impact of construction materials of monocrystalline and perovskite photovoltaic power plants towards their sustainable development.

The research object was the materials and construction elements of two 1 MW photovoltaic power plants. The first one was based on modules made of monocrystalline silicon, while the second one was based on perovskite (tandem type). The analyses were carried out using the Life Cycle Assessment method (IM-PACT World+ model, IPCC and CED). The assessment was carried out separately for five sets of elements: support structure, photovoltaic panels, inverter station, electrical installation and transformer. Additionally, two post-consumer management scenarios were adopted: landfill and recycling.

In the literature, there are few studies on the life cycle of photovoltaic power plants. Most often, the life cycle of photovoltaic modules or only the materials from which the cells are manufactured is assessed. The studies usually concern silicon technologies, mainly single-crystalline silicon [8,9,10,11,12,13,14,15,16], and multi-crystalline silicon [11,13,16,17,18,19,20,21,22], less often—amorphous-silicon [16,19,20], and multi-junction thin-film silicon [23]. Individual works were devoted to cells made of other materials, e.g., cadmium telluride [13,20,22], copper-indium-gallium-diselenide [23], and DSSC [24]. There are also a small number of articles addressing the life cycle of systems with large installed capacity [9,25,26]. Studies conducted so far have usually not included more detailed analyses of the impacts of photovoltaic power plants, which may pose a threat to human health, cause a decrease in the quality of ecosystems or deepen the depletion of raw material resources.

The achievement of the main objective of the study allowed for the formulation of the following conclusions:

− From the perspective of the life cycle of materials and elements of both analyzed photovoltaic power plants, the categories of impact with the highest level of harmful impact on human health include water availability, human health, and climate change, human health, long term. In terms of impact on ecosystem quality, these are the categories of freshwater ecotoxicity, long term, and climate change, ecosystem quality, long-term (Table 1 and Table 2, Figure 11, Figure 12, Figure 13 and Figure 14).
− Among the processes related to the reduction in water availability in the life cycles of both power plants (impact category: water availability, human health), one can distinguish, first, the consumption of water from various sources (lakes, rivers, wells, etc.) and its use for cooling turbines (during electricity generation).
− The chemical compounds affecting climate change (impact category: climate change, human health, long term, and climate change, ecosystem quality, long term) in the life cycles of both power plants, characterized by the highest harmful impact values include emissions of carbon dioxide, methane, dinitrogen monoxide, sulfur hexafluoride, tetrafluoromethane (CFC-14), hexafluoroethane (HFC-116) and trifluoromethane (HFC-23).
− Among the construction materials of the analyzed power plants, the life cycle of which has the greatest impact on the increase in freshwater ecotoxicity (impact category: freshwater ecotoxicity, long term), the following can be distinguished: copper, aluminum, iron, strontium, nickel, zinc, manganese, cadmium and vanadium.
− In the case of both assessed power plants, the highest level of total harmful impact on human health and ecosystem quality was observed during the life cycle of photovoltaic panels (Figure 14 and Figure 15).
− The life cycle of a sc-Si power plant is associated with higher environmental costs incurred due to the deterioration of human health and the reduction in the quality of the ecosystem compared to the life cycle of a PSC power plant (Table 4).
− Both post-consumer management processes in the form of storage and recycling cause higher environmental costs in the area of human health than in the area of ecosystem quality (Table 4).
− The element of both assessed power plants with the highest environmental life cycle cost are photovoltaic panels (Figure 16).
− For all elements, the environmental cost of their life cycle is lower in the case of a photovoltaic power plant based on the use of perovskite panels and for the form of post-consumer management using recycling processes (Figure 16).
− The total environmental cost of the life cycle of a sc-Si power plant is higher than that of a PSC power plant (Figure 17).
− The highest level of greenhouse gas emissions in the life cycles of both power plants is due to the use of fossil sources (fuels and mineral raw materials, impact category: GWP100—fossil) (Table 5).
− The highest GHG emissions in the life cycles of both power plants are characteristic of photovoltaic panels (Figure 18).
− For all assessed electoral elements, the use of recycling processes is associated with lower greenhouse gas emissions compared to landfill management (Figure 18).
− The life cycle of a power plant using monocrystalline silicon panels is characterized by higher GHG emissions than a power plant using perovskite panels (Figure 19).
− The majority of energy in the life cycles of both examined photovoltaic power plants comes from non-renewable sources (impact category: non-renewable, fossil), mainly from crude oil, coal and gas (Table 6).
− Photovoltaic panels are the element of the power plant that stands out with the highest cumulative demand for energy (Figure 20).
− The life cycle of a sc-Si photovoltaic power plant is characterized by a higher total cumulative energy demand compared to a PSC power plant (Figure 21).
− The use of recycling processes enables a significant reduction in the energy demand in the life cycles of both tested technical facilities (Figure 21).
− Photovoltaic power plants based on perovskite modules fit better into the main assumptions of sustainable development than power plants using monocrystalline silicon panels.

The increasing share of renewable energy sources (including photovoltaic power plants) in the global energy balance enables more sustainable and economically efficient use of fossil fuels. It also improves the condition of the environment and increases the level of energy security. An additional advantage is more dynamic regional development, the creation of new jobs and a reduction in many environmental problems [96,97,98].

Continuous technological and scientific development allows for the implementation of the best available techniques (BAT). They should take into account the minimization of energy and material consumption from the perspective of the entire life cycle of photovoltaic power plants. Nowadays, it is necessary to change the way of managing environmental resources towards the rationalization of the use of natural resources. In order to achieve this goal, more sustainable technologies should be popularized, not only in the area of renewable energy sources, but in all sectors of the economy [99,100].

New methods of producing and recycling plastics, materials and components of photovoltaic power plants are being developed all over the world in order to reduce the negative impact of this type of waste on the environment and create the possibility of recovering at least some of its value (especially from photovoltaic panels that are no longer suitable for further use). Today, recycling methods allow for the recovery of only a certain part of the materials used, so there is a lot of room for progress in this area. Reducing energy and material consumption in the life cycles of photovoltaic power plants translates into a reduction in the level of their harmful impact on human health, environmental quality and the depletion of raw materials. Life cycle studies of innovative construction materials that better fit into the assumptions of sustainable development can have a significant impact on policy and future industrial practices. They provide a basis for developing recommendations in the area of sustainable, efficient development of the renewable energy sector, and, in particular,—photovoltaics. Identification of areas in the life cycle of photovoltaic power plants with the potentially greatest harmful impact on the environment will enable taking action to reduce material and energy consumption and the harmful emissions of processes related to the production, operational and post-consumer management of their construction materials. It will also facilitate work on creating innovative, more environmentally friendly materials and elements. The search for an optimal solution for a photovoltaic power plant will, therefore, primarily consist of finding the right structure that will allow obtaining the desired quality of a technical object and determining process parameters that guarantee the lowest energy and material costs at each stage of its life cycle.

## Figures and Tables

**Table 1:** Characterization of environmental consequences in the life cycle of a photovoltaic power plant based on monocrystalline (sc-Si) modules for considered impact categories and different post-consumer management scenarios (model IMPACT World+ Endpoint ver. 1.02).

Element of a Technical Object	Support Structure		Photovoltaic Panels		Inverter Station		Electrical Installation		Transformer		Unit
Form of Post-Use Management	Landfill	Recycling	Landfill	Recycling	Landfill	Recycling	Landfill	Recycling	Landfill	Recycling	
Impact Category											
Climate change, human health, short term	3.11 × 10^−5^	2.81 × 10^−6^	4.70 × 10^−3^	3.17 × 10^−3^	6.29 × 10^−5^	5.66 × 10^−5^	2.67 × 10^−5^	1.68 × 10^−5^	4.53 × 10^−5^	4.51 × 10^−5^	DALY
**Climate change, human health, long term**	**9.42 × 10^−5^**	**−7.62 × 10^−6^**	**2.47 × 10^−2^**	**1.82 × 10^−2^**	**1.88 × 10^−4^**	**1.34 × 10^−4^**	**8.25 × 10^−5^**	**4.07 × 10^−5^**	**1.31 × 10^−4^**	**1.09 × 10^−4^**	**DALY**
Photochemical oxidant formation	4.15 × 10^−7^	1.49 × 10^−7^	1.35 × 10^−5^	8.19 × 10^−7^	6.95 × 10^−7^	5.14 × 10^−7^	3.96 × 10^−7^	2.03 × 10^−7^	4.93 × 10^−7^	4.19 × 10^−7^	DALY
Ionizing radiation, human health	1.66 × 10^−7^	−1.12 × 10^−7^	1.34 × 10^−5^	9.33 × 10^−6^	2.96 × 10^−7^	2.41 × 10^−7^	9.49 × 10^−8^	4.72 × 10^−8^	1.96 × 10^−7^	1.29 × 10^−6^	DALY
Ozone layer depletion	7.13 × 10^−9^	−4.50 × 10^−9^	9.47 × 10^−7^	2.73 × 10^−7^	1.41 × 10^−8^	5.10 × 10^−9^	5.38 × 10^−9^	2.04 × 10^−9^	1.14 × 10^−8^	3.51 × 10^−9^	DALY
Human toxicity cancer, short term	8.10 × 10^−5^	9.38 × 10^−6^	6.12 × 10^−5^	3.93 × 10^−4^	1.40 × 10^−4^	7.37 × 10^−5^	6.16 × 10^−5^	7.35 × 10^−6^	1.17 × 10^−4^	8.58 × 10^−5^	DALY
Human toxicity cancer, long term	3.49 × 10^−6^	2.19 × 10^−6^	2.22 × 10^−5^	1.51 × 10^−5^	9.99 × 10^−6^	4.66 × 10^−6^	1.68 × 10^−6^	5.88 × 10^−6^	4.03 × 10^−6^	1.85 × 10^−6^	DALY
Human toxicity non-cancer, short term	1.71 × 10^−5^	4.00 × 10^−5^	7.51 × 10^−5^	4.24 × 10^−5^	2.92 × 10^−5^	4.49 × 10^−5^	1.30 × 10^−5^	3.41 × 10^−5^	2.33 × 10^−5^	3.56 × 10^−5^	DALY
Human toxicity non-cancer, long term	2.01 × 10^−4^	1.37 × 10^−4^	7.06 × 10^−4^	4.16 × 10^−5^	3.43 × 10^−4^	1.58 × 10^−4^	1.47 × 10^−4^	1.07 × 10^−4^	2.76 × 10^−4^	1.13 × 10^−4^	DALY
Particulate matter formation	5.80 × 10^−5^	1.60 × 10^−5^	3.02 × 10^−3^	2.08 × 10^−3^	1.15 × 10^−4^	6.09 × 10^−5^	4.05 × 10^−5^	1.73 × 10^−5^	6.75 × 10^−5^	4.03 × 10^−5^	DALY
**Water availability, human health**	**1.89 × 10^−2^**	**−1.72 × 10^−2^**	**2.13 × 10^0^**	**1.27 × 10^0^**	**3.10 × 10^−2^**	**1.03 × 10^−2^**	**1.14 × 10^−2^**	**2.16 × 10^−3^**	**2.54 × 10^−2^**	**1.01 × 10^−2^**	**DALY**
Climate change, ecosystem quality, short term	7.70 × 10^−8^	6.94 × 10^−9^	2.16 × 10^−5^	1.46 × 10^−5^	1.36 × 10^−7^	1.22 × 10^−7^	5.77 × 10^−8^	3.63 × 10^−8^	9.80 × 10^−8^	9.73 × 10^−8^	PDF × m^2^ × yr
**Climate change, ecosystem quality, long term**	**2.37 × 10^−7^**	**−1.90 × 10^−8^**	**6.62 × 10^−5^**	**4.56 × 10^−5^**	**4.14 × 10^−7^**	**2.94 × 10^−7^**	**1.81 × 10^−7^**	**8.97 × 10^−8^**	**2.89 × 10^−7^**	**2.39 × 10^−7^**	**PDF × m^2^ × yr**
Marine acidification, short term	5.89 × 10^−9^	7.53 × 10^−10^	1.72 × 10^−6^	1.19 × 10^−6^	1.06 × 10^−8^	7.66 × 10^−9^	4.61 × 10^−9^	2.29 × 10^−9^	7.43 × 10^−9^	6.14 × 10^−9^	PDF × m^2^ × yr
Marine acidification, long term	5.42 × 10^−8^	6.94 × 10^−9^	1.59 × 10^−5^	1.09 × 10^−5^	9.81 × 10^−8^	7.06 × 10^−8^	4.25 × 10^−8^	2.11 × 10^−8^	6.84 × 10^−8^	5.66 × 10^−8^	PDF × m^2^ × yr
Freshwater ecotoxicity, short term	1.59 × 10^−8^	1.33 × 10^−8^	4.05 × 10^−6^	2.05 × 10^−6^	3.37 × 10^−8^	1.73 × 10^−8^	9.92 × 10^−9^	3.62 × 10^−9^	2.09 × 10^−8^	1.47 × 10^−8^	PDF × m^2^ × yr
**Freshwater ecotoxicity, long term**	**5.12 × 10^−5^**	**2.31 × 10^−5^**	**1.58 × 10^−3^**	**6.78 × 10^−4^**	**1.45 × 10^−4^**	**1.56 × 10^−5^**	**4.17 × 10^−5^**	**1.13 × 10^−6^**	**6.01 × 10^−5^**	**9.27 × 10^−6^**	**PDF × m^2^ × yr**
Freshwater acidification	1.34 × 10^−8^	2.14 × 10^−9^	8.80 × 10^−7^	5.91 × 10^−7^	3.97 × 10^−8^	1.66 × 10^−8^	1.05 × 10^−8^	3.78 × 10^−9^	1.68 × 10^−8^	7.59 × 10^−9^	PDF × m^2^ × yr
Terrestrial acidification	8.64 × 10^−8^	1.50 × 10^−8^	5.74 × 10^−6^	3.87 × 10^−6^	2.46 × 10^−7^	1.05 × 10^−7^	7.78 × 10^−8^	2.83 × 10^−8^	1.05 × 10^−7^	4.84 × 10^−8^	PDF × m^2^ × yr
Freshwater eutrophication	1.18 × 10^−10^	−8.07 × 10^−11^	6.19 × 10^−9^	2.59 × 10^−9^	2.29 × 10^−10^	1.95 × 10^−10^	7.55 × 10^−11^	3.63 × 10^−11^	1.57 × 10^−10^	1.22 × 10^−10^	PDF × m^2^ × yr
Marine eutrophication	2.10 × 10^−9^	1.48 × 10^−9^	1.27 × 10^−7^	8.17 × 10^−8^	3.68 × 10^−9^	2.59 × 10^−9^	1.45 × 10^−9^	1.37 × 10^−9^	2.69 × 10^−9^	1.93 × 10^−9^	PDF × m^2^ × yr
Ionizing radiation, ecosystem quality	1.80 × 10^−16^	−9.66 × 10^−17^	2.22 × 10^−14^	1.45 × 10^−14^	3.23 × 10^−16^	1.48 × 10^−16^	1.66 × 10^−16^	3.82 × 10^−17^	2.09 × 10^−16^	7.89 × 10^−17^	PDF × m^2^ × yr
Land transformation, biodiversity	4.32 × 10^−8^	3.38 × 10^−9^	4.64 × 10^−6^	2.77 × 10^−6^	8.48 × 10^−8^	5.84 × 10^−8^	3.65 × 10^−8^	2.28 × 10^−8^	5.27 × 10^−8^	3.39 × 10^−8^	PDF × m^2^ × yr
Land occupation, biodiversity	1.29 × 10^−8^	7.19 × 10^−9^	2.15 × 10^−6^	1.49 × 10^−6^	2.58 × 10^−8^	1.84 × 10^−8^	1.16 × 10^−8^	7.18 × 10^−9^	1.51 × 10^−8^	1.10 × 10^−8^	PDF × m^2^ × yr
Water availability, freshwater ecosystem	1.95 × 10^−10^	−1.58 × 10^−10^	2.37 × 10^−9^	1.09 × 10^−9^	3.51 × 10^−10^	6.55 × 10^−12^	1.50 × 10^−10^	9.90 × 10^−12^	2.69 × 10^−10^	8.04 × 10^−12^	PDF × m^2^ × yr
Water availability, terrestrial ecosystem	1.49 × 10^−11^	6.07 × 10^−12^	3.80 × 10^−9^	2.32 × 10^−9^	2.65 × 10^−11^	1.79 × 10^−11^	1.15 × 10^−11^	5.80 × 10^−12^	1.73 × 10^−11^	1.26 × 10^−11^	PDF × m^2^ × yr
Thermally polluted water	1.13 × 10^−12^	5.26 × 10^−13^	5.19 × 10^−10^	3.30 × 10^−10^	2.60 × 10^−12^	1.41 × 10^−12^	1.07 × 10^−12^	3.26 × 10^−13^	2.19 × 10^−12^	1.92 × 10^−12^	PDF × m^2^ × yr

**Bold data**—the highest levels of negative consequences.

**Table 2:** Characterization of environmental consequences in the life cycle of a perovskite photovoltaic power plant (PSC) for considered impact categories and different post-consumer management scenarios (model IMPACT World+ Endpoint ver. 1.02).

Element of a Technical Object	Support Structure		Photovoltaic Panels		Inverter Station		Electrical Installation		Transformer		Unit
Form of Post-Use Management	Landfill	Recycling	Landfill	Recycling	Landfill	Recycling	Landfill	Recycling	Landfill	Recycling	
Impact Category											
Climate change, human health, short term	2.82 × 10^−5^	2.54 × 10^−6^	4.26 × 10^−3^	2.87 × 10^−3^	5.69 × 10^−5^	5.12 × 10^−5^	2.41 × 10^−5^	1.52 × 10^−5^	4.10 × 10^−5^	4.08 × 10^−5^	DALY
**Climate change, human health, long term**	**8.52 × 10^−5^**	**−6.90 × 10^−6^**	**2.23 × 10^−2^**	**1.64 × 10^−2^**	**1.70 × 10^−4^**	**1.21 × 10^−4^**	**7.46 × 10^−5^**	**3.68 × 10^−5^**	**1.19 × 10^−4^**	**9.85 × 10^−5^**	**DALY**
Photochemical oxidant formation	3.76 × 10^−7^	1.35 × 10^−7^	1.22 × 10^−5^	7.41 × 10^−7^	6.29 × 10^−7^	4.65 × 10^−7^	3.58 × 10^−7^	1.84 × 10^−7^	4.46 × 10^−7^	3.79 × 10^−7^	DALY
Ionizing radiation, human health	1.50 × 10^−7^	−1.02 × 10^−7^	1.22 × 10^−5^	8.44 × 10^−6^	2.68 × 10^−7^	2.18 × 10^−7^	8.59 × 10^−8^	4.27 × 10^−8^	1.78 × 10^−7^	1.17 × 10^−7^	DALY
Ozone layer depletion	6.45 × 10^−9^	−4.08 × 10^−9^	8.57 × 10^−7^	2.47 × 10^−7^	1.27 × 10^−8^	4.62 × 10^−9^	4.87 × 10^−9^	1.85 × 10^−9^	1.03 × 10^−8^	3.18 × 10^−9^	DALY
Human toxicity cancer, short term	7.33 × 10^−5^	8.48 × 10^−6^	5.53 × 10^−4^	3.56 × 10^−4^	1.26 × 10^−4^	6.67 × 10^−5^	5.58 × 10^−5^	6.65 × 10^−6^	1.06 × 10^−4^	7.77 × 10^−5^	DALY
Human toxicity cancer, long term	3.16 × 10^−6^	1.98 × 10^−6^	2.01 × 10^−5^	1.36 × 10^−5^	9.04 × 10^−6^	4.22 × 10^−6^	1.52 × 10^−6^	5.32 × 10^−7^	3.65 × 10^−6^	1.67 × 10^−6^	DALY
Human toxicity non-cancer, short term	1.55 × 10^−4^	3.62 × 10^−5^	6.80 × 10^−4^	3.84 × 10^−4^	2.64 × 10^−4^	4.06 × 10^−5^	1.18 × 10^−4^	3.08 × 10^−5^	2.11 × 10^−4^	3.22 × 10^−5^	DALY
Human toxicity non-cancer, long term	1.81 × 10^−4^	1.24 × 10^−4^	6.39 × 10^−4^	3.76 × 10^−5^	3.11 × 10^−4^	1.43 × 10^−4^	1.33 × 10^−4^	9.65 × 10^−5^	2.50 × 10^−4^	1.02 × 10^−4^	DALY
Particulate matter formation	5.25 × 10^−5^	1.45 × 10^−5^	2.74 × 10^−3^	1.88 × 10^−3^	1.04 × 10^−4^	5.51 × 10^−5^	3.66 × 10^−5^	1.57 × 10^−5^	6.10 × 10^−5^	3.65 × 10^−5^	DALY
**Water availability, human health**	**1.71 × 10^−2^**	**−1.55 × 10^−2^**	**1.92 × 10^0^**	**1.15 × 10^0^**	**2.80 × 10^−2^**	**9.36 × 10^−3^**	**1.03 × 10^−2^**	**1.95 × 10^−3^**	**2.29 × 10^−2^**	**9.10 × 10^−3^**	**DALY**
Climate change, ecosystem quality, short term	6.97 × 10^−8^	6.28 × 10^−9^	1.95 × 10^−5^	1.32 × 10^−5^	1.23 × 10^−7^	1.11 × 10^−7^	5.22 × 10^−8^	3.28 × 10^−8^	8.87 × 10^−8^	8.81 × 10^−8^	PDF × m^2^ × yr
**Climate change, ecosystem quality, long term**	**2.14 × 10^−7^**	**−1.72 × 10^−8^**	**5.99 × 10^−5^**	**4.12 × 10^−5^**	**3.74 × 10^−7^**	**2.66 × 10^−7^**	**1.64 × 10^−7^**	**8.12 × 10^−8^**	**2.61 × 10^−7^**	**2.16 × 10^−7^**	**PDF × m^2^ × yr**
Marine acidification, short term	5.33 × 10^−9^	6.81 × 10^−10^	1.56 × 10^−6^	1.07 × 10^−6^	9.63 × 10^−9^	6.93 × 10^−9^	4.17 × 10^−9^	2.07 × 10^−9^	6.72 × 10^−9^	5.55 × 10^−9^	PDF × m^2^ × yr
Marine acidification, long term	4.91 × 10^−8^	6.28 × 10^−9^	1.44 × 10^−5^	9.90 × 10^−6^	8.88 × 10^−8^	6.39 × 10^−8^	3.84 × 10^−8^	1.91 × 10^−8^	6.19 × 10^−8^	5.12 × 10^−8^	PDF × m^2^ × yr
Freshwater ecotoxicity, short term	1.44 × 10^−8^	1.20 × 10^−8^	3.67 × 10^−6^	1.85 × 10^−6^	3.05 × 10^−8^	1.56 × 10^−8^	8.97 × 10^−9^	3.27 × 10^−9^	1.89 × 10^−8^	1.33 × 10^−8^	PDF × m^2^ × yr
**Freshwater ecotoxicity, long term**	**4.63 × 10^−5^**	**2.09 × 10^−5^**	**1.43 × 10^−3^**	**6.14 × 10^−4^**	**1.32 × 10^−4^**	**1.41 × 10^−5^**	**3.78 × 10^−5^**	**1.03 × 10^−6^**	**5.44 × 10^−5^**	**8.39 × 10^−6^**	**PDF × m^2^ × yr**
Freshwater acidification	1.21 × 10^−8^	1.93 × 10^−9^	7.96 × 10^−7^	5.35 × 10^−7^	3.59 × 10^−8^	1.50 × 10^−8^	9.53 × 10^−9^	3.42 × 10^−9^	1.52 × 10^−8^	6.87 × 10^−9^	PDF × m^2^ × yr
Terrestrial acidification	7.82 × 10^−8^	1.35 × 10^−8^	5.19 × 10^−6^	3.50 × 10^−6^	2.23 × 10^−7^	9.54 × 10^−8^	7.04 × 10^−8^	2.56 × 10^−8^	9.48 × 10^−8^	4.38 × 10^−8^	PDF × m^2^ × yr
Freshwater eutrophication	1.07 × 10^−10^	−7.30 × 10^−11^	5.60 × 10^−9^	2.34 × 10^−9^	2.08 × 10^−10^	1.77 × 10^−10^	6.83 × 10^−11^	3.28 × 10^−11^	1.42 × 10^−10^	1.10 × 10^−10^	PDF × m^2^ × yr
Marine eutrophication	1.90 × 10^−9^	1.34 × 10^−9^	1.15 × 10^−7^	7.39 × 10^−8^	3.33 × 10^−9^	2.35 × 10^−9^	1.31 × 10^−9^	1.24 × 10^−9^	2.43 × 10^−9^	1.75 × 10^−9^	PDF × m^2^ × yr
Ionizing radiation, ecosystem quality	1.63 × 10^−16^	−8.74 × 10^−17^	2.00 × 10^−14^	1.31 × 10^−14^	2.92 × 10^−16^	1.34 × 10^−16^	1.50 × 10^−16^	3.45 × 10^−17^	1.89 × 10^−16^	7.14 × 10^−17^	PDF × m^2^ × yr
Land transformation, biodiversity	3.91 × 10^−8^	3.06 × 10^−9^	4.20 × 10^−6^	2.51 × 10^−6^	7.67 × 10^−8^	5.28 × 10^−8^	3.30 × 10^−8^	2.06 × 10^−8^	4.77 × 10^−8^	3.07 × 10^−8^	PDF × m^2^ × yr
Land occupation, biodiversity	1.16 × 10^−8^	6.51 × 10^−9^	1.94 × 10^−6^	1.35 × 10^−6^	2.33 × 10^−8^	1.66 × 10^−8^	1.05 × 10^−8^	6.50 × 10^−9^	1.37 × 10^−8^	9.99 × 10^−9^	PDF × m^2^ × yr
Water availability, freshwater ecosys.	1.76 × 10^−10^	−1.43 × 10^−10^	2.14 × 10^−9^	9.82 × 10^−10^	3.18 × 10^−10^	5.93 × 10^−12^	1.35 × 10^−10^	8.96 × 10^−12^	2.43 × 10^−10^	7.28 × 10^−12^	PDF × m^2^ × yr
Water availability, terrestrial ecosys.	1.35 × 10^−11^	5.49 × 10^−12^	3.44 × 10^−9^	2.10 × 10^−9^	2.40 × 10^−11^	1.62 × 10^−11^	1.04 × 10^−11^	5.25 × 10^−12^	1.57 × 10^−11^	1.14 × 10^−11^	PDF × m^2^ × yr
Thermally polluted water	1.02 × 10^−12^	4.76 × 10^−13^	4.70 × 10^−10^	2.99 × 10^−10^	2.35 × 10^−12^	1.28 × 10^−12^	9.72 × 10^−13^	2.95 × 10^−13^	1.98 × 10^−12^	1.73 × 10^−12^	PDF × m^2^ × yr

**Bold data**—the highest levels of negative consequences.

**Table 3:** Characterization of environmental consequences in the life cycle of the studied photovoltaic power plants for the considered impact areas and different post-consumer development scenarios (model IMPACT World+ Endpoint ver. 1.02).

Area of Influence	Power Plant Components	Support Structure		Photovoltaic Panels		Inverter Station		Electrical Installation		Transformer		Unit
	Post-Use Development Scenario	Landfill	Recycling	Landfill	Recycling	Landfill	Recycling	Landfill	Recycling	Landfill	Recycling	
Human health	**sc-Si**	**1.96 × 10^−2^**	**−1.70 × 10^−2^**	**2.16 × 10^0^**	**1.29 × 10^0^**	**3.21 × 10^−2^**	**1.09 × 10^−2^**	**1.19 × 10^−2^**	**2.38 × 10^−3^**	**2.62 × 10^−2^**	**1.05 × 10^−2^**	DALY
	PSC	1.77 × 10^−2^	−1.54 × 10^−2^	1.95 × 10^0^	1.17 × 10^0^	2.91 × 10^−2^	9.84 × 10^−3^	1.08 × 10^−2^	2.16 × 10^−3^	2.37 × 10^−2^	9.49 × 10^−3^	
Ecosystem quality	**sc-Si**	**5.18 × 10^−5^**	**2.32 × 10^−5^**	**1.70 × 10^−3^**	**7.62 × 10^−4^**	**1.47 × 10^−4^**	**1.63 × 10^−5^**	**4.22 × 10^−5^**	**1.35 × 10^−6^**	**6.08 × 10^−5^**	**9.79 × 10^−6^**	PDF × m^2 ×^ yr
	PSC	4.68 × 10^−5^	2.10 × 10^−5^	1.54 × 10^−3^	6.89 × 10^−4^	1.33 × 10^−4^	1.47 × 10^−5^	3.81 × 10^−5^	1.22 × 10^−6^	5.50 × 10^−5^	8.86 × 10^−6^	

**Bold data**—the highest levels of negative consequences.

**Table 4:** Grouping and weighing environmental consequences in the life cycle of the studied photovoltaic power plants for the considered impact areas and different post-consumer development scenarios (model IMPACT World+ Endpoint ver. 1.02, unit: EUR).

Element of a Technical Object		Support Structure		Photovoltaic Panels		Inverter Station		Electrical Installation		Transformer		Unit
Form of Post-Use Management		Landfill	Recycling	Landfill	Recycling	Landfill	Recycling	Landfill	Recycling	Landfill	Recycling	
Areas of Influence	Type of Power Plant											
Human health	**sc-Si**	**1.24 × 10^5^**	**2.51 × 10^4^**	**7.27 × 10^6^**	**4.03 × 10^6^**	**3.03 × 10^5^**	**1.13 × 10^5^**	**8.11 × 10^4^**	**2.37 × 10^4^**	**1.50 × 10^5^**	**6.28 × 10^4^**	**EUR**
	PSC	1.23 × 10^5^	7.74 × 10^4^	3.80 × 10^6^	2.06 × 10^6^	1.94 × 10^5^	4.59 × 10^4^	1.04 × 10^5^	3.71 × 10^4^	1.36 × 10^5^	4.43 × 10^4^	EUR
Ecosystem quality	**sc-Si**	**1.23 × 10^5^**	**7.74 × 10^4^**	**3.80 × 10^6^**	**2.06 × 10^6^**	**1.94 × 10^5^**	**4.59 × 10^4^**	**1.04 × 10^5^**	**3.71 × 10^4^**	**1.36 × 10^5^**	**4.43 × 10^4^**	**EUR**
	PSC	1.01 × 10^5^	6.30 × 10^4^	1.57 × 10^6^	7.96 × 10^5^	1.75 × 10^5^	4.16 × 10^4^	8.28 × 10^4^	2.95 × 10^4^	1.23 × 10^5^	4.01 × 10^4^	EUR

**Bold data**—the highest levels of negative consequences.

**Table 5:** Characterization of environmental consequences for greenhouse gas emissions in the life cycle of the studied photovoltaic power plants for the considered impact categories and different post-consumer management scenarios (model IPCC 2021 GWP100 ver. 1.01, unit: kg CO_2_ eq).

Element of a Technical Object		Support Structure		Photovoltaic Panels		Inverter Station		Electrical Installation		Transformer		Unit
Form of Post-Use Management		Landfill	Recycling	Landfill	Recycling	Landfill	Recycling	Landfill	Recycling	Landfill	Recycling	
Impact Category	Type of Power Plant											
**GWP100—fossil**	**sc-Si**	**1.32 × 10^4^**	**−3.97 × 10^2^**	**1.01 × 10^6^**	**5.51 × 10^5^**	**2.32 × 10^4^**	**6.75 × 10^3^**	**8.76 × 10^3^**	**1.76 × 10^3^**	**1.83 × 10^4^**	**6.10 × 10^3^**	**kg CO_2_ eq**
	**PSC**	**1.07 × 10^4^**	**−1.55 × 10^3^**	**5.45 × 10^5^**	**2.69 × 10^5^**	**2.10 × 10^4^**	**6.11 × 10^3^**	**6.98 × 10^3^**	**1.40 × 10^3^**	**1.66 × 10^4^**	**5.52 × 10^3^**	**kg CO_2_ eq**
GWP100—biogenic	sc-Si	2.86 × 10^2^	9.02 × 10^1^	4.06 × 10^3^	2.22 × 10^3^	4.60 × 10^2^	3.78 × 10^1^	2.04 × 10^2^	2.13 × 10^0^	3.68 × 10^2^	4.67 × 10^1^	kg CO_2_ eq
	PSC	2.33 × 10^2^	5.58 × 10^1^	2.28 × 10^3^	1.10 × 10^3^	4.16 × 10^2^	3.42 × 10^1^	1.62 × 10^2^	1.70 × 10^0^	3.33 × 10^2^	4.23 × 10^1^	kg CO_2_ eq
GWP100—land transformation	sc-Si	4.33 × 10^1^	1.48 × 10^1^	1.95 × 10^3^	9.95 × 10^2^	6.03 × 10^1^	9.60 × 10^0^	3.31 × 10^1^	1.20 × 10^0^	4.84 × 10^1^	1.11 × 10^1^	kg CO_2_ eq
	PSC	3.53 × 10^1^	9.51 × 10^0^	1.02 × 10^3^	4.74 × 10^2^	5.45 × 10^1^	8.69 × 10^0^	2.64 × 10^1^	9.56 × 10^−1^	4.38 × 10^1^	1.01 × 10^1^	kg CO_2_ eq

**Bold data**—the highest levels of negative consequences.

**Table 6:** Characterization of energy demand in the life cycle of the studied photovoltaic power plants for the considered impact categories and different post-consumer management scenarios (model CED—Cumulative Energy Demand ver. 1.11, unit: MJ).

Element of a Technical Object		Support Structure		Photovoltaic Panels		Inverter Station		Electrical Installation		Transformer		Unit
Form of Post-Use Management		Landfill	Recycling	Landfill	Recycling	Landfill	Recycling	Landfill	Recycling	Landfill	Recycling	
Impact Category	Type of Power Plant											
**Non-renewable,** **fossil**	**sc-Si**	**1.84 × 10^5^**	**1.69 × 10^4^**	**1.27 × 10^7^**	**6.96 × 10^6^**	**2.76 × 10^5^**	**1.23 × 10^5^**	**1.52 × 10^5^**	**4.98 × 10^4^**	**2.16 × 10^5^**	**1.08 × 10^5^**	**MJ**
	**PSC**	**1.43 × 10^5^**	**1.31 × 10^4^**	**6.81 × 10^6^**	**5.23 × 10^6^**	**2.38 × 10^5^**	**1.06 × 10^5^**	**1.15 × 10^5^**	**3.78 × 10^4^**	**1.86 × 10^5^**	**9.34 × 10^4^**	**MJ**
Non-renewable, nuclear	sc-Si	2.55 × 10^4^	−2.18 × 10^4^	1.86 × 10^6^	9.60 × 10^5^	4.05 × 10^4^	2.11 × 10^4^	2.26 × 10^4^	7.45 × 10^3^	3.24 × 10^4^	1.57 × 10^4^	MJ
	PSC	1.98 × 10^4^	−1.69 × 10^4^	1.07 × 10^6^	7.79 × 10^5^	3.49 × 10^4^	1.82 × 10^4^	1.72 × 10^4^	5.65 × 10^3^	2.79 × 10^4^	1.35 × 10^4^	MJ
Non-renewable, biomass	sc-Si	2.11 × 10^1^	5.08 × 10^0^	9.71 × 10^2^	1.90 × 10^2^	2.95 × 10^1^	5.27 × 10^0^	1.58 × 10^1^	7.61 × 10^−1^	2.56 × 10^1^	6.74 × 10^0^	MJ
	PSC	1.64 × 10^1^	3.94 × 10^0^	4.85 × 10^2^	1.21 × 10^2^	2.54 × 10^1^	4.54 × 10^0^	1.20 × 10^1^	5.77 × 10^−1^	2.21 × 10^1^	5.81 × 10^0^	MJ
Renewable, biomass	sc-Si	3.39 × 10^3^	1.44 × 10^3^	5.73 × 10^5^	3.07 × 10^5^	4.57 × 10^3^	2.13 × 10^3^	2.96 × 10^3^	1.10 × 10^3^	3.84 × 10^3^	2.02 × 10^3^	MJ
	PSC	2.63 × 10^3^	1.12 × 10^3^	3.03 × 10^5^	2.29 × 10^5^	3.94 × 10^3^	1.84 × 10^3^	2.25 × 10^3^	8.32 × 10^2^	3.31 × 10^3^	1.74 × 10^3^	MJ
Renewable, wind, solar, geothe	sc-Si	3.69 × 10^3^	1.95 × 10^3^	3.72 × 10^5^	1.53 × 10^5^	5.95 × 10^3^	2.71 × 10^3^	2.80 × 10^3^	7.28 × 10^2^	4.20 × 10^3^	2.29 × 10^3^	MJ
	PSC	2.86 × 10^3^	1.51 × 10^3^	2.09 × 10^5^	1.22 × 10^5^	5.12 × 10^3^	2.34 × 10^3^	2.12 × 10^3^	5.52 × 10^2^	3.62 × 10^3^	1.98 × 10^3^	MJ
Renewable, water	sc-Si	3.25 × 10^4^	−2.03 × 10^4^	2.71 × 10^6^	2.09 × 10^6^	9.78 × 10^4^	8.70 × 10^3^	1.98 × 10^4^	9.07 × 10^2^	4.16 × 10^4^	5.19 × 10^3^	MJ
	PSC	4.19 × 10^4^	−2.62 × 10^4^	4.92 × 10^6^	3.00 × 10^6^	1.14 × 10^5^	1.01 × 10^4^	2.61 × 10^4^	1.20 × 10^3^	4.83 × 10^4^	6.03 × 10^3^	MJ

**Bold data**—the highest levels of negative consequences.

**Table 7:** Comparison of the total environmental impact of the life cycles of a photovoltaic power plant based on monocrystalline silicon (sc-Si) and perovskite (PSC) modules, for two post-consumer development scenarios (landfill and recycling).

Area of Influence	sc-Si		PSC		Unit
	Landfill	Recycling	Landfill	Recycling	
Human health	**2.25 × 10^0^**	1.30 × 10^0^	2.04 × 10^0^	1.17 × 10^0^	DALY
Ecosystem quality	**2.00 × 10^−3^**	8.12 × 10^−4^	1.81 × 10^−3^	7.35 × 10^−4^	PDF × m^2^ × yr
Human health	**7.93 × 10^6^**	4.25 × 10^6^	4.36 × 10^6^	2.27 × 10^6^	EUR
Ecosystem quality	**4.36 × 10^6^**	2.27 × 10^6^	2.05 × 10^6^	9.70 × 10^5^	
Total impact	**1.23 × 10^7^**	6.52 × 10^6^	6.59 × 10^6^	3.81 × 10^6^	
GWP100	**1.08 × 10^6^**	5.69 × 10^5^	6.05 × 10^5^	2.82 × 10^5^	kg CO_2_eq
Cumulative energy demand	**2.16 × 10^7^**	1.17 × 10^7^	1.21 × 10^7^	8.73 × 10^6^	MJ

**Bold data**—the highest levels of negative consequences.

## Data Availability

The data presented in this study are available on request from the author.

## References

[1] Li C., Chen L., Jiang F., Song Z., Wang X., Balvanz A., Ugur E., Liu Y., Liu C., Maxwell A. (2024). Diamine chelates for increased stability in mixed Sn–Pb and all-perovskite tandem solar cells. Nat. Energy.

[2] Demirel Y. (2016). Energy: Production, Conversion, Storage, Conservation and Coupling.

[3] Singh R., Kumar S. (2017). Green Technologies and Environmental Sustainability.

[4] Monge J. Global Solar PV Installed Capacity Will More Than Triple in the Next Ten Years. https://www.woodmac.com/news/opinion/global-solar-PV-installed-more-than-triple-in-the-next-ten-years/.

[5] Andersen O. (2013). Unintended Consequences of Renewable Energy.

[6] Dincer I., Midilli A., Kucuk H. (2014). Progress in Sustainable Energy Technologies: Generating Renewable Energy.

[7] Leda P., Idzikowski A., Piasecka I., Baldowska-Witos P., Cierlicki T., Zawada M. (2023). Management of Environmental Life Cycle Impact Assessment of a Photovoltaic Power Plant on the Atmosphere, Water, and Soil Environment. Energies.

[8] Wang X., Tian X., Chen X., Ren L., Geng C. (2022). A Review of End-of-Life Crystalline Silicon Solar Photovoltaic Panel Recycling Technology. Sol. Energy Mater. Sol. Cells.

[9] Kato K., Murata A., Sakuta K. (2017). An evaluation on the life cycle of photovoltaic energy system considering production energy of off-grade silicon. Sol. Energy Mat. Sol. Cells.

[10] Golroudbary S.R., Lundström M., Wilson B.P. (2024). Analogical Environmental Cost Assessment of Silicon Flows Used in Solar Panels by the US and China. Sci. Rep..

[11] Dones R., Frischknecht R. (2018). Life cycle assessment of photovoltaic systems: Results of Swiss studies on energy chains. Prog. Photovolt. Res. Appl..

[12] Heath G.A., Silverman T.J., Kempe M., Deceglie M., Ravikumar D., Remo T., Cui H., Sinha P., Libby C., Shaw S. (2020). Research and Development Priorities for Silicon Photovoltaic Module Recycling to Support a Circular Economy. Nat. Energy.

[13] Fthenakis V.M., Kim H.C. (2007). Greenhouse-gas emissions from solar electric and nuclear power: A life-cycle study. Energy Policy.

[14] Reich N.H., Alsema E.A., van Sark W.G.J.H.M., Turkenburg W.C., Sinke W.C. (2011). Greenhouse Gas Emissions Associated with Photovoltaic Electricity from Crystalline Silicon Modules under Various Energy Supply Options. Prog. Photovolt. Res. Appl..

[15] Frankl P., Masini A., Gamberale M., Toccaceli D. (2018). Simplified life-cycle analysis of PV systems in buildings: Present situation and future trends. Prog. Photovolt. Res. Appl..

[16] Alsema E.A. (2000). Energy pay-back time and CO_2_ emissions of PV systems. Prog. Photovolt. Res. Appl..

[17] Oliver M., Jackson T. (2000). The evolution of economic and environmental cost for crystalline silicon photovoltaics. Energy Policy.

[18] Nomura N., Inaba A., Tonooka Y., Akai M. (2001). Life-cycle emission of oxidic gases from power-generation systems. Appl. Energy.

[19] Kato K., Murata A., Sakuta K. (2018). Energy pay-back time and lifecycle CO_2_ emission of residential PV power system with silicon PV module. Prog. Photovolt. Res. Appl..

[20] Ito M., Kato K., Komoto K., Kichimi T., Kurokawa K. (2008). A comparative study on cost and life-cycle analysis for 100 MW very large-scale PV (VLS-PV) systems in deserts using m-Si, a-Si, CdTe, and CIS modules. Prog. Photovolt. Res. Appl..

[21] Ito M., Kato K., Sugihara H., Kichimi T., Song J., Kurokawa K. (2003). A preliminary study on potential for very large scale photovoltaic power generation (VLS-PV) system in the Gobi desert from economic and environmental viewpoints. Sol. Energy Mat. Sol. Cells.

[22] Fthenakis V.M., Alsema E. (2006). Photovoltaics energy payback times, greenhouse gas emissions and external costs: 2004—Early 2005 status. Prog. Photovolt. Res. Appl..

[23] Bravi M., Parisi M.L., Tiezzi E., Basosi R. (2011). Life cycle assessment of a micro morph photovoltaic system. Energy.

[24] Greijer H., Karlson L., Lindquist S.E., Hagfeldt A. (2001). Environmental aspects of electricity generation from a nanocrystalline dye sensitized solar cell system. Renew. Energy.

[25] Kato K., Hibino T., Komoto K., Ihara S., Yamamoto S., Fujihara H. (2001). A life-cycle analysis on thin-film CdS/CdTe PV modules. Sol. Energy Mat. Sol. Cells.

[26] Schaefer H., Hagedorn G. (2002). Hidden energy and correlated environmental characteristics of PV power generation. Renew. Energy.

[27] Manser J.S., Christians J.A., Kamat V.P. (2016). Intriguing Optoelectronic Properties of Metal Halide Perovskites. Chem. Rev..

[28] Kojima A., Teshima K., Shirai Y., Miyasaka T. (2009). Organometal Halide Perovskites as Visible-Light Sensitizers for Photovoltaic Cells. J. Am. Chem. Soc..

[29] Sun K., Wang Y., Xu H., Zhang J., Zhu Y., Hu Z. (2019). Short-Term Stability of Perovskite Solar Cells Affected by In Situ Interface Modification. Sol. RRL.

[30] (2006). Environmental Management—Life Cycle Assessment—Principles and Framework (Edition 2).

[31] (2006). Environmental management—Life Cycle Assessment—Requirements and Guidelines (Edition 1).

[32] Piotrowska K., Piasecka I., Gola A., Kosicka E., Hamrol A., Grabowska M. (2024). Assessment of the Impact of Selected Segments of Road Transport on the Natural Environment Using LCA Analysis. Advances in Manufacturing IV. MANUFACTURING 2024. Lecture Notes in Mechanical Engineering.

[33] Muthu S.S. (2021). Life Cycle Sustainability Assessment (LCSA).

[34] Schmidt W.P. (2024). Solutions for Sustainability Challenges. Technical Sustainability Management and Life Cycle Thinking.

[35] Nakamura S. (2023). A Practical Guide to Industrial Ecology by Input-Output Analysis.

[36] Mroziński A., Piasecka I. (2015). Selected Aspects of Building, Operation and Environmental Impact of Offshore Wind Power Electric Plants. Pol. Marit. Res..

[37] Hesser F., Kral I., Obersteiner G., Hörtenhuber S., Kühmaier M., Zeller V., Schebek L. (2023). Progress in Life Cycle Assessment 2021.

[38] Siwiec D., Pacana A. (2024). Decision-Making Model Supporting Eco-Innovation in Energy Production Based on Quality, Cost and Life Cycle Assessment (LCA). Energies.

[39] Stoffels P., Kaspar J., Baehre D., Vielhaber M. (2017). Holistic Material Selection Approach for More Sustainable Products. Procedia Manuf..

[40] Reap J., Roman F., Duncan S., Bras B. (2008). A survey of unresolved problems in life cycle assessment. Int. J. Life Cycle Assess..

[41] Sobocińska M., Mazurek-Łopacińska K., Graczyk A., Kociszewski K., Krupowicz J. (2022). Decision-Making Processes of Renewable Energy Consumers Compared to Other Categories of Ecological Products. Energies.

[42] Majid S., Zhang X., Khaskheli M.B., Hong F., King P.J.H., Shamsi I.H. (2023). Eco-Efficiency, Environmental and Sustainable Innovation in Recycling Energy and Their Effect on Business Performance: Evidence from European SMEs. Sustainability.

[43] Kłos Z. (2002). Ecobalancial assessment of chosen packaging processes in food industry. Int. J. Life Cycle Assess..

[44] Klemeš J.J., Varbanov P.S., Ocłoń P., Chin H.H. (2019). Towards Efficient and Clean Process Integration: Utilisation of Renewable Resources and Energy-Saving Technologies. Energies.

[45] Varun, Bhat I.K., Prakash R. (2009). LCA of Renewable Energy for Electricity Generation Systems—A Review. Renew. Sustain. Energy Rev..

[46] Kjaer L.L., Pigosso D.C.A., McAloone T.C., Birkved M. (2018). Guidelines for evaluating the environmental performance of Product/Service-Systems through life cycle assessment. J. Clean. Prod..

[47] Suppipat S., Chotiratanapinun T., Teachavorasinskun K., Hu A.H. (2023). Design for Enhancing Eco-Efficiency of Energy-Related Products. The Integration of Simplified LCA Tools in Industrial Design Education.

[48] Piotrowska K., Piasecka I., Kasner R. (2022). Assessment of the Life Cycle of a Wind and Photovoltaic Power Plant in the Context of Sustainable Development of Energy Systems. Materials.

[49] Wang S., Su D. (2022). Sustainable Product Innovation and Consumer Communication. Sustainability.

[50] Proske M., Finkbeiner M. (2020). Obsolescence in LCA–Methodological Challenges and Solution Approaches. Int. J. Life Cycle Assess..

[51] Palousis N., Luong L., Abhary K. (2008). An Integrated LCA/LCC Framework for Assessing Product Sustainability Risk.

[52] Finkbeiner M., Inaba A., Tan R., Christiansen K., Klüppel H.-J. (2006). The New International Standards for Life Cycle Assessment: ISO 14040 and ISO 14044. Int. J. Life Cycle Assess..

[53] Piasecka I., Tomporowski A. (2018). Analysis of Environmental and Energetical Possibilities of Sustainable Development of Wind and Photovoltaic Power Plants. Probl. Sustain. Dev..

[54] Kutraleeswaran M., Venkatachalam M., Saroja M., Gowthaman M., Shankar S. (2017). Dye sensitized solar cells—A review. Trans. Indian Ceram. Soc..

[55] Tian X., Stranks S.D., You F. (2020). Life cycle energy use and environmental implications of high-performance perovskite tandem solar cells. Sci. Adv..

[56] Finnveden G., Hauschild M.Z., Ekvall T., Guinée J., Heijungs R., Hellweg S., Koehler A., Pennington D., Suh S. (2009). Recent developments in Life Cycle Assessment. J. Environ. Manag..

[57] Guinée J. (2002). Handbook on Life Cycle Assessment: Operational Guide to the ISO Standards.

[58] Fukushige S., Kobayashi H., Yamasue E., Hara K. (2024). EcoDesign for Sustainable Products, Services and Social Systems II.

[59] Bałdowska-Witos P., Piasecka I., Flizikowski J., Tomporowski A., Idzikowski A., Zawada M. (2021). Life Cycle Assessment of Two Alternative Plastics for Bottle Production. Materials.

[60] Hauschild M.Z., Goedkoop M., Guinée J., Heijungs R., Huijbregts M., Jolliet O., Margni M., De Schryver A., Humbert S., Laurent A. (2013). Identifying best existing practice for characterization modeling in life cycle impact assessment. Int. J. Life Cycle Assess..

[61] Bare J.C., Hofstetter P., Pennington D.W., Udo de Haes H.A. (2000). Midpoints versus endpoints: The sacrifices and benefits. Int. J. Life Cycle Assess..

[62] Jolliet O., Müller-Wenk R., Bare J., Brent A., Goedkoop M., Heijungs R., Itsubo N., Peña C., Pennington D., Potting J. (2004). The LCIA midpoint-damage framework of the UNEP/SETAC life cycle initiative. Int. J. Life Cycle Assess..

[63] Portillo F., Alcayde A., Garcia R.M., Fernandez-Ros M., Gazquez J.A., Novas N. (2024). Life Cycle Assessment in Renewable Energy: Solar and Wind Perspectives. Environments.

[64] Piasecka I., Tomporowski A., Piotrowska K. (2018). Environmental analysis of post-use management of car tires. Przem. Chem..

[65] Millet D., Bistagnino L., Lanzavecchia C., Camous R., Poldma T. (2007). Does the Potential of the Use of LCA Match the Design Team Needs?. J. Clean. Prod..

[66] Su D. (2020). Sustainable Product Development. Tools, Methods and Examples.

[67] Cooper J.S. (2003). Specifying functional units and reference flows for comparable alternatives. Int. J. Life Cycle Assess..

[68] Finkbeiner M. (2014). Product environmental footprint—Breakthrough or breakdown for policy implementation of life cycle assessment?. Int. J. Life Cycle Assess..

[69] Sobaszek L., Piasecka I., Flizikowski J., Tomporowski A., Sokolovskij E., Baldowska-Witos P. (2023). Environmentally Oriented Analysis of Benefits and Expenditures in the Life Cycle of a Wind Power Plant. Materials.

[70] Heijungs R. (2024). Probability, Statistics and Life Cycle Assessment. Guidance for Dealing with Uncertainty and Sensitivity.

[71] Bulle C., Margni M., Patouillard L., Boulay A.M., Bourgault G., De Bruille V., Cao V., Hauschild M., Henderson A., Humbert S. (2019). IMPACT World+: A globally regionalized life cycle impact assessment method. Int. J. Life Cycle Assess..

[72] Jolliet O., Frischknecht R., Bare J., Boulay A.M., Bulle C., Fantke P., Gheewala S., Hauschild M., Itsubo N., Margni M. (2014). Global guidance on environmental life cycle impact assessment indicators: Findings of the scoping phase. Int. J. Life Cycle Assess..

[73] Nalau J., Gilmore E., Howden M. (2024). Improving adaptation assessment in the IPCC. NJP Clim. Action.

[74] Masson-Delmotte V., Zhai P., Pirani A., Connors S.L., Péan C., Berger S., Caud N., Chen Y., Goldfarb L., Gomis M.I. (2021). IPCC, 2021: Climate Change 2021: The Physical Science Basis. Contribution of Working Group I to the Sixth Assessment Report of the Intergovernmental Panel on Climate Change Cambridge.

[75] Muñoz I., Schmidt J.H. (2016). Methane oxidation, biogenic carbon, and the IPCC’s emission metrics. Proposal for a consistent greenhouse-gas accounting. Int. J. Life Cycle Assess..

[76] Piotrowska K., Piasecka I. (2021). Specification of Environmental Consequences of the Life Cycle of Selected Post-Production Waste of Wind Power Plants Blades. Materials.

[77] Frischknecht R., Wyss F., Büsser Knöpfel S., Lützkendorf T., Balouktsi M. (2015). Cumulative energy demand in LCA: The energy harvested approach. Int. J. Life Cycle Assess..

[78] Patel M. (2003). Cumulative energy demand (CED) and cumulative CO_2_ emissions for products of the organic chemical industry. Energy.

[79] Huijbregts M.A.J., Hellweg S., Frischknecht R., Hendriks H.W.M., Hungerbühler K., Hendriks A.J. (2010). Cumulative Energy Demand As Predictor for the Environmental Burden of Commodity Production. Environ. Sci. Technol..

[80] Kłos Z., Kalkowska J., Kasprzak J. (2022). Towards a Sustainable Future—Life Cycle Management. Challenges and Prospects.

[81] Curran M.A. (2023). Interpretation, Critical Review and Reporting in Life Cycle Assessment.

[82] Piasecka I., Bałdowska-Witos P., Piotrowska K., Kruszelnicka W., Flizikowski J., Tomporowski A. (2021). Ecological life cycle assessment of the 1 MW photovoltaic power plant under Polish environmental conditions. Przem. Chem..

[83] Koehler A. (2008). Water use in LCA: Managing the planet’s freshwater resources. Int. J. Life Cycle Assess..

[84] Boulay A.M., Bulle C., Bayart J.B., Deschênes L., Margni M. (2011). Regional Characterization of Freshwater Use in LCA: Modeling Direct Impacts on Human Health. Environ. Sci. Technol..

[85] Pfister S., Koehler A., Hellweg S. (2009). Assessing the Environmental Impacts of Freshwater Consumption in LCA. Environ. Sci. Technol..

[86] Andrew R.M. (2020). A comparison of estimates of global carbon dioxide emissions from fossil carbon sources. Earth Syst. Sci. Data.

[87] Burke P.J., Shahiduzzaman M., Stern D.I. (2015). Carbon dioxide emissions in the short run: The rate and sources of economic growth matter. Glob. Environ. Chang..

[88] Georgopoulos P.G., Roy A., Yonone-Lioy M.J., Opiekun R.E., Lioy P.J. (2011). Environmental copper: Its dynamics and human exposure issues. J. Toxicol. Environ. Health.

[89] Ibrahim M., El-Haes H. (2005). Computational spectroscopic study of copper, cadmium, lead and zinc interactions in the environment. Int. J. Environ. Pollut..

[90] Laws E.A. (2013). Environmental Toxicology.

[91] Mar K.A., Unger C., Walderdorff L., Butler T. (2022). Beyond CO_2_ equivalence: The impacts of methane on climate, ecosystems, and health. Environ. Sci. Policy.

[92] Miller S.M., Wofsy S.C., Michalak A.M., Kort E.A., Andrews A.E., Biraud S.C., Dlugokencky E.J., Eluszkiewicz J., Fischer M.L., Janssens-Maenhout G. (2013). Anthropogenic emissions of methane. Earth Atmos. Planet. Sci..

[93] Piotrowska K., Bałdowska-Witos P., Piasecka I., Kasner R., Kruszelnicka W., Tomporowski A. (2021). Identification of the most important areas of environmental impact over the life cycle of car tires. Przem. Chem..

[94] El Chaar L., Lamont L.A., El Zein N. (2011). Review of photovoltaic technologies. Renew. Sustain. Energy Rev..

[95] Zacher L.W. (2017). Technology, Society and Sustainability: Selected Concepts, Issues and Cases.

[96] Bagnall D.M., Boreland M. (2008). Photovoltaic technologies. Energy Policy.

[97] Khan I. (2022). Renewable Energy and Sustainability.

[98] Leda P., Kruszelnicka W., Leda A., Piasecka I., Kłos Z., Tomporowski A., Flizikowski J., Opielak M. (2024). Life Cycle Analysis of a Photovoltaic Power Plant Using the CED Method. Energies.

[99] Rigatos G.G. (2016). Intelligent Renewable Energy Systems: Modelling and Control.

[100] Ross J.R.H. (2022). Sustainable Energy.

